# Gut-derived *Enterococcus faecium* from ulcerative colitis patients promotes colitis in a genetically susceptible mouse host

**DOI:** 10.1186/s13059-019-1879-9

**Published:** 2019-11-25

**Authors:** Jun Seishima, Noriho Iida, Kazuya Kitamura, Masahiro Yutani, Ziyu Wang, Akihiro Seki, Taro Yamashita, Yoshio Sakai, Masao Honda, Tatsuya Yamashita, Takashi Kagaya, Yukihiro Shirota, Yukako Fujinaga, Eishiro Mizukoshi, Shuichi Kaneko

**Affiliations:** 1 Department of Gastroenterology, Graduate School of Medical Sciences, Kanazawa University, Kanazawa, Ishikawa 920-8641 Japan; 2 Department of Bacteriology, Graduate School of Medicinal Sciences, Kanazawa University, Kanazawa, Ishikawa Japan; 3 Department of Advanced Medical Technology, Graduate School of Health Medicine, Kanazawa University, Kanazawa, Ishikawa Japan

**Keywords:** Inflammatory bowel disease, Crohn’s disease, Microbiota, Metagenome

## Abstract

**Background:**

Recent metagenomic analyses have revealed dysbiosis of the gut microbiota of ulcerative colitis (UC) patients. However, the impacts of this dysbiosis are not fully understood, particularly at the strain level.

**Results:**

We perform whole-genome shotgun sequencing of fecal DNA extracts from 13 healthy donors and 16 UC and 8 Crohn’s disease (CD) patients. The microbiota of UC and CD patients is taxonomically and functionally divergent from that of healthy donors, with *E. faecium* being the most differentially abundant species between the two microbial communities. Transplantation of feces from UC or CD patients into *Il10*^−/−^ mice promotes pathological inflammation and cytokine expression in the mouse colon, although distinct cytokine expression profiles are observed between UC and CD. Unlike isolates derived from healthy donors, *E. faecium* isolates from the feces of UC patients, along with *E. faecium* strain ATCC 19434, promotes colitis and colonic cytokine expression. Inflammatory *E. faecium* strains, including ATCC 19434 and a UC-derived strain, cluster separately from commercially available probiotic strains based on whole-genome shotgun sequencing analysis. The presence of *E. faecium* in fecal samples is associated with large disease extent and the need for multiple medications in UC patients.

**Conclusions:**

*E. faecium* strains derived from UC patients display an inflammatory genotype that causes colitis.

## Background

The hundreds of trillions of microbes in the gastrointestinal tract contribute to many host physiological processes including nutrient acquisition and development of the gut immune system, while dysbiosis of the microbiota can contribute to the development of several diseases [1]. The commensal gut microbiota is closely related to the pathogenesis of inflammatory bowel diseases (IBD), including ulcerative colitis (UC) and Crohn’s disease (CD) [2]. A significant amount of information on the role of the gut microbiota in IBD patients has been accumulated based on clinical studies, metagenome analyses, and animal experiments. The recent development of high-throughput metagenome sequencing techniques and analysis methods has uncovered dysbiosis of the microbiota of IBD patients, with a decrease in microbial diversity compared with healthy individuals [3–5]. In addition, the gut community of IBD patients shows an increased prevalence of the phylum *Proteobacteria* [6], including inflammatory species *Escherichia coli* [4], and a decrease in the phylum *Firmicutes* [6], including anti-inflammatory species *Faecalibacterium prausnitzii* [4, 7]. While metagenomic analysis can reveal an association between dysbiosis and disease, animal studies can demonstrate a causative association between specific bacteria and the pathogenesis of colitis. According to previous mouse studies, *F. prausnitzii* [8], *Bacteroides fragilis* [9], and *Clostridia* species [10] attenuate intestinal inflammation via various mechanisms, including induction of IL-10 and regulatory T cells in the colon, whereas *Escherichia coli* [11] and *Enterococcus faecalis* [11, 12] promote colitis in mouse models.

Although previous studies have built up a significant body of information, questions remain about the causal relationship between human IBD pathogenesis and microbiota dysbiosis. Firstly, because of the complexity of the gut microbiota and the multifactorial nature of IBD, it is unclear exactly which bacterial species in the dysbiotic IBD microbiota are responsible for the pathogenesis of colitis. Because few reports reproduce the microbial composition of the dysbiotic microbiota in colitic mouse models, it is unclear whether decreases or increases in a single anti-inflammatory or pro-inflammatory species are truly responsible for IBD pathogenesis. Secondly, the specific genotypes of bacterial species putatively responsible for IBD pathogenesis are yet to be elucidated. Metagenomic analysis of the gut microbiota, particularly 16S ribosomal RNA (rRNA) sequencing-based approaches, cannot identify the composition of the microbiota at the strain level. Therefore, it is difficult to judge based only on metagenomic analysis whether the decreased or increased prevalence of a species in the IBD microbiota has a pathogenic effect.

In the present study, to answer these questions, we attempted to find a link between human metagenome data and phenotype in a mouse model of colitis. We first analyzed whole-genome shotgun sequencing data corresponding to fecal metagenomes obtained from patients with IBD. Feces from the same subjects was then transplanted into *Il10*^−/−^ mice to assess the causal relationship between dysbiosis of the microbiota and intestinal inflammation. Finally, the genotype of a bacterial species possibly responsible for inflammation, *Enterococcus faecium*, was identified by sequencing multiple strains isolated from UC patients. Thus, we clearly showed a causal relationship between UC-derived bacterial strains and colonic inflammation.

## Results

### The gut microbiota of IBD patients is taxonomically and functionally divergent from that of healthy donors

To investigate taxonomic and functional differences in gut microbiota between patients with IBD and healthy controls, fecal samples were collected from 13 healthy donors (HD), 16 UC patients, and 8 CD patients. The CD patients were younger and had higher serum C-reactive protein levels than the HD, although no difference was observed between HD and UC patients in other baseline characteristics (Additional file 1: Table S1). The extent of disease in UC patients was either left-sided colitis or pancolitis (left-sided colitis, 68.8%; pancolitis, 31.3%; Additional file 1: Table S2). Fifteen of the 16 UC patients were being treated with medications, including mesalazine, corticosteroids, tacrolimus, azathioprine, mercaptopurine, and TNF antagonist, and both active and inactive colitis patients were represented in the UC cohort according to total Mayo scores (average Mayo score, 3.25; Additional file 1: Table S2). All the CD patients were being treated with medications, including mesalazine, corticosteroids, azathioprine, and TNF antagonist, and in most cases, the disease was inactive based on the Crohn’s disease activity index (average CDAI, 71.1; Additional file 1: Table S3).

**Fig. 1:**
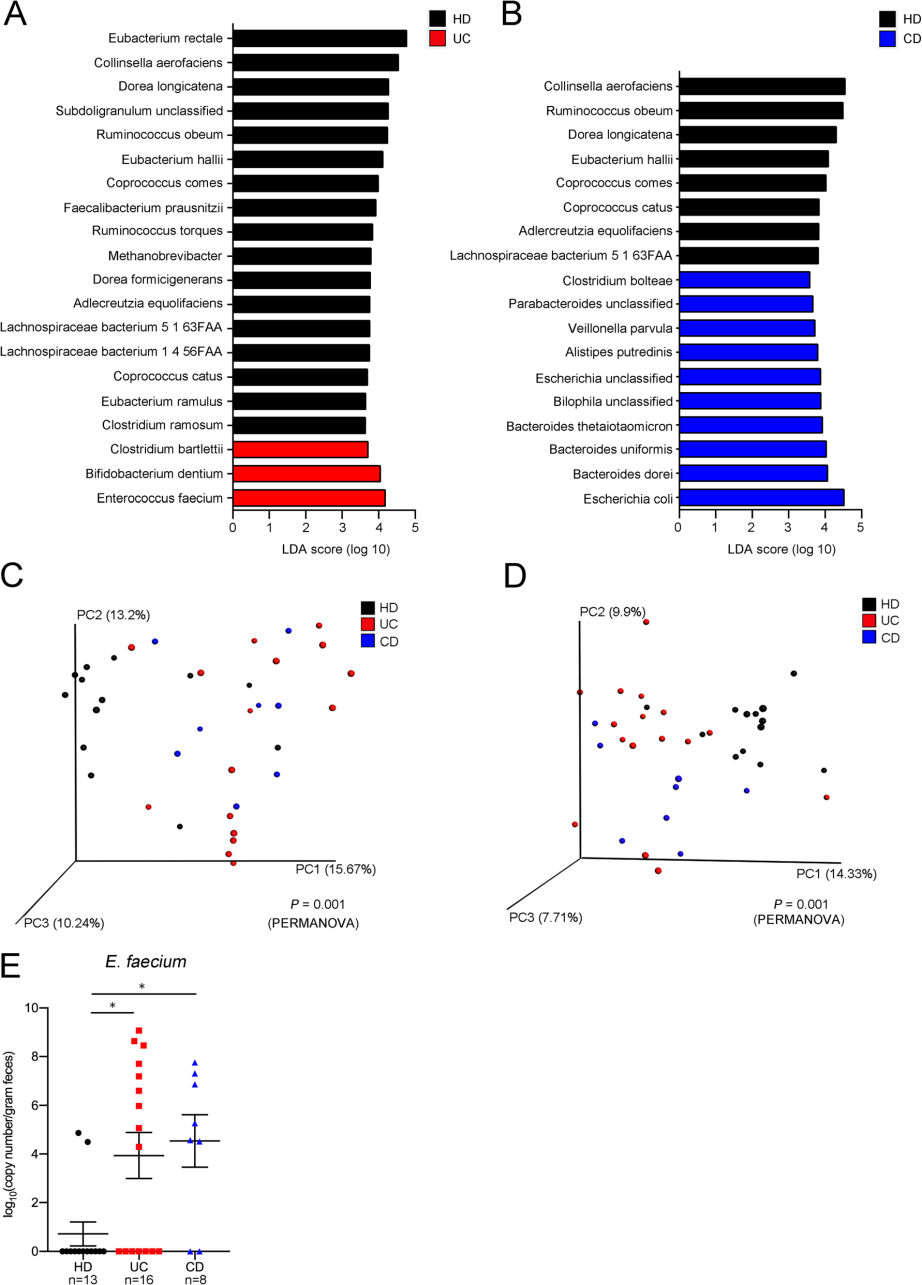
The microbiota of inflammatory bowel disease (IBD) patients is taxonomically and functionally divergent from that of healthy donor (HD) subjects. **a** Linear discriminative analysis (LDA) was performed using linear discriminative analysis effect size (LEfSe) to identify significant differences in relative abundance of various taxonomic groups in the feces of HD subjects compared with ulcerative colitis (UC) patients, or **b** HD patients compared with Crohn’s disease (CD) patients. Differentially abundant genera for which the corresponding LDA scores indicate *P* < 0.05 are shown in the graphs. **c** Distances between the patient groups were calculated using the Bray-Curtis index based on taxonomic data or **d** Kyoto Encyclopedia of Genes and Genomes (KEGG) pathway data and visualized by principal coordinate analysis. Significant differences between groups were determined by PERMANOVA, and *P* values are shown at the bottom of the plots. **e** Copy number of *Enterococcus faecium* in feces was determined by PCR. *P < 0.05

Whole-genome shotgun sequencing of fecal DNA samples revealed significant differences in abundance of 20 and 18 bacterial species between the HD and UC, and HD and CD communities, respectively (Fig. 1a, b). Of these species, *E. faecium* and *Escherichia coli* had the highest linear discriminative analysis (LDA) scores in the UC and CD samples, respectively, compared with the HD samples (Fig. 1a, b). Anti-inflammatory species *F. prausnitzii* was less abundant in the UC communities compared with the HD communities in this analysis (Fig. 1a), as previously reported [7]. Seven species, including *Collinsella aerofaciens*, *Ruminococcus obeum*, *Dorea longicatena*, *Eubacterium hallii*, *Coprococcus comes*, *Adlercreutzia equolifaciens*, and a *Lachnospiraceae* sp. bacterium, were less abundant in both the UC and CD communities compared with the HD community (Fig. 1a, b). Metabolic pathway analysis of the gut microbiota revealed that 38 and 35 Kyoto Encyclopedia of Genes and Genomes (KEGG) pathways were significantly different between the HD and UC, or HD and CD samples, respectively (Additional file 1: Tables S4 and S5). Pathways involved in glycerophospholipid metabolism were less abundant in both the UC and CD microbiota compared with those of the HD patients (Additional file 1: Tables S4 and S5). Analysis of the UC microbiota showed that pathways involved in DNA replication and repair, including base excision repair and mismatch repair, were more abundant compared with the microbiota of the HD (Additional file 1: Table S4). In β-diversity analysis and principal coordinate analysis (PCoA), the UC and CD microbiota showed taxonomically (Fig. 1c) and functionally (Fig. 1d) differential plots compared with the HD microbiota (*P* = 0.001, PERMANOVA). Because the abundance of *E. faecium* in UC patients has not previously been reported, the difference in abundance of fecal *E. faecium* between HD and UC patients was confirmed by quantitative PCR, which is more sensitive in detection than metagenomic sequencing analysis (Fig. 1e). Thus, these results confirm both taxonomic and functional differences in the gut microbiota of UC and CD patients compared with the HD.

### The gut microbiota of UC and CD patients cause colitis in *Il10*^−/−^ mice

To explore a causal relationship between the gut microbiota and colitis, the fecal samples used in the sequencing analysis were transplanted into *Il10*^−/−^ colitis-susceptible mice. Fecal microbiota samples from each of the subjects were transplanted into at least two microbiota-depleted *Il10*^−/−^ mice, and colonic inflammation was evaluated on day 28 post-transplantation by pathology and gene expression analyses. Although longitudinal analysis of the fecal microbiota following fecal transplantation revealed that the composition of the human gut community was not completely restored in the transplanted mice (Additional file 2: Figure S1a and b), the composition of the murine microbiota was uniquely dependent on the human subjects’ microbiota and remained stably segregated from the composition in other mice up to 28 days post-transplantation (Additional file 2: Figure S1c, *P* = 0.001, PERMANOVA). Effects of cage difference (Additional file 2: Figure S1d) and repeats of transplantation (Additional file 2: Figure S1e) were modest, and PCoA plots were significantly differentially segregated by differences between the donor feces (Additional file 2: Figure S1d, *P* = 0.001; Additional file 2: Figure S1e, *P* = 0.029: PERMANOVA). Therefore, we evaluated the effects of these unique human-derived microbial communities on the severity of colitis in the mouse model.

**Fig. 2:**
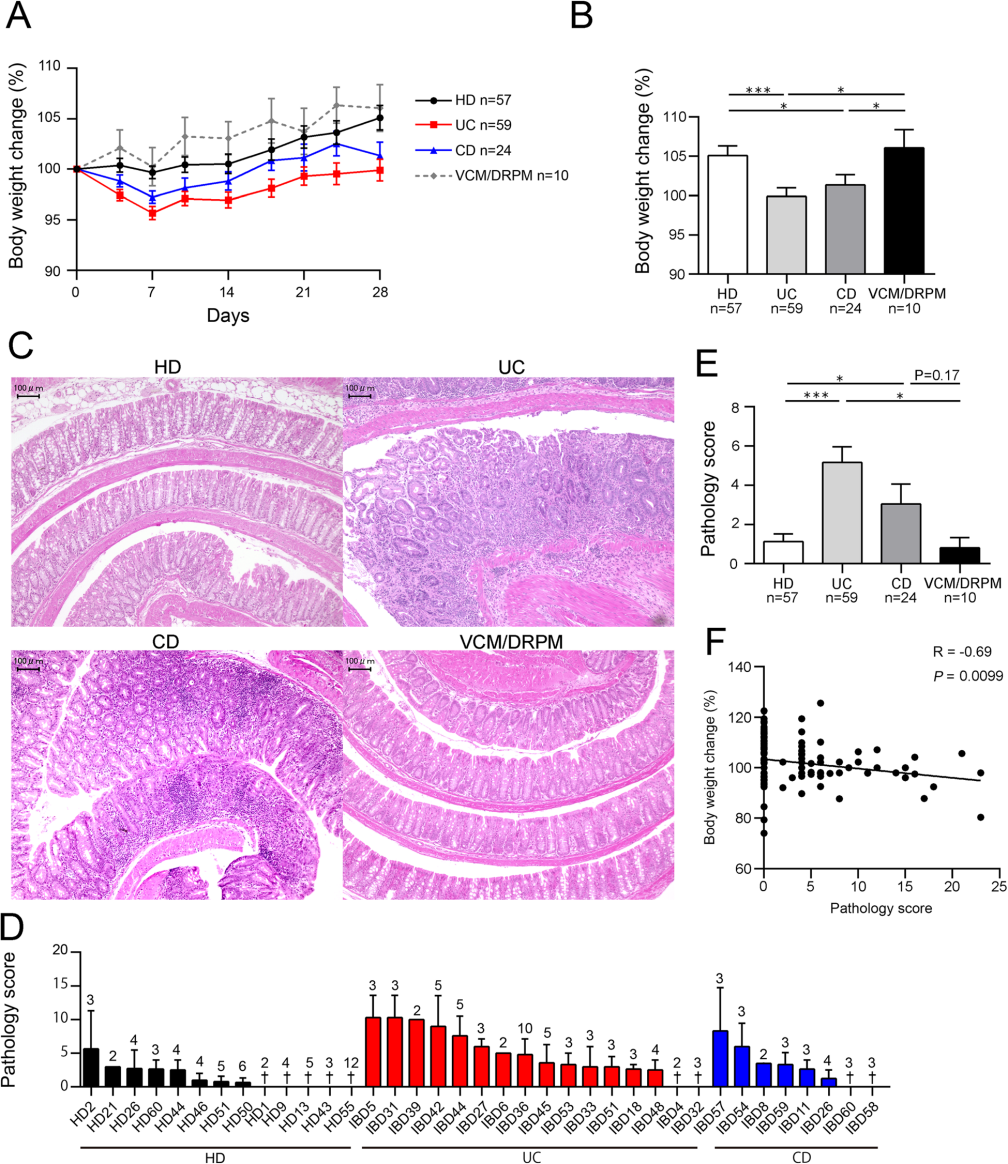
The fecal microbiota of IBD patients cause colitis in *Il10*^−/−^ mice. Fecal suspensions from HD, UC, or CD subjects were transplanted into microbiota-depleted *Il10*^−/−^ mice. The control group was treated with antibiotics (vancomycin/doripenem; VCM/DRPM) in the absence of transplantation. All mice were euthanized 28 days post-transplantation. **a** Changes in body weight (%) of each treatment group throughout the course of the experiment and **b** on day 28. **c** Representative histological sections of the murine colon at the time of euthanasia. Bars, 100 μm. **d** Mean pathology scores of mice corresponding to individual fecal donors or **e** complete treatment groups. †, average pathology score of 0. The identification codes of individual subjects are shown on the *x*-axis in **d**. **f** Linear regression line determined from plots showing average changes in body weight (%) on day 28 and pathology scores of mice in all groups. The regression coefficient (*R*) and *P* value are shown. Values shown in **a**, **b**, **d**, and **e** are the mean ± SE. The numbers above the error bars in **d** indicate the number of mice in each category. Statistical differences between two values were analyzed using a Mann-Whitney *U* test. *P < 0.05; **P < 0.01; ***P < 0.001

Body weight of mice transplanted with the UC or the CD patient microbiota increased less than that of mice transplanted with the HD microbiota or the microbiota-depleted controls (Fig. 2a, b). Pathological changes were more severe in rectum segments than in the proximal colon in this mouse model (Additional file 2: Figure S2). A greater number of mice transplanted with the UC or CD microbiota showed epithelial hyperplasia with lymphoplasmacytosis, obliteration of normal architecture, and erosion, particularly in the rectal segments, compared with the HD group and microbiota-depleted controls (Fig. 2c and Additional file 2: Figure S2). The average pathology score for the colon was significantly higher for the UC and CD microbiota-transplanted mice compared with the HD microbiota-transplanted mice (Fig. 2d, e). In addition, body weight change was negatively correlated with pathology score (Fig. 2f), implying that inflammation in the colon was responsible for the decreased body weight.

**Fig. 3:**
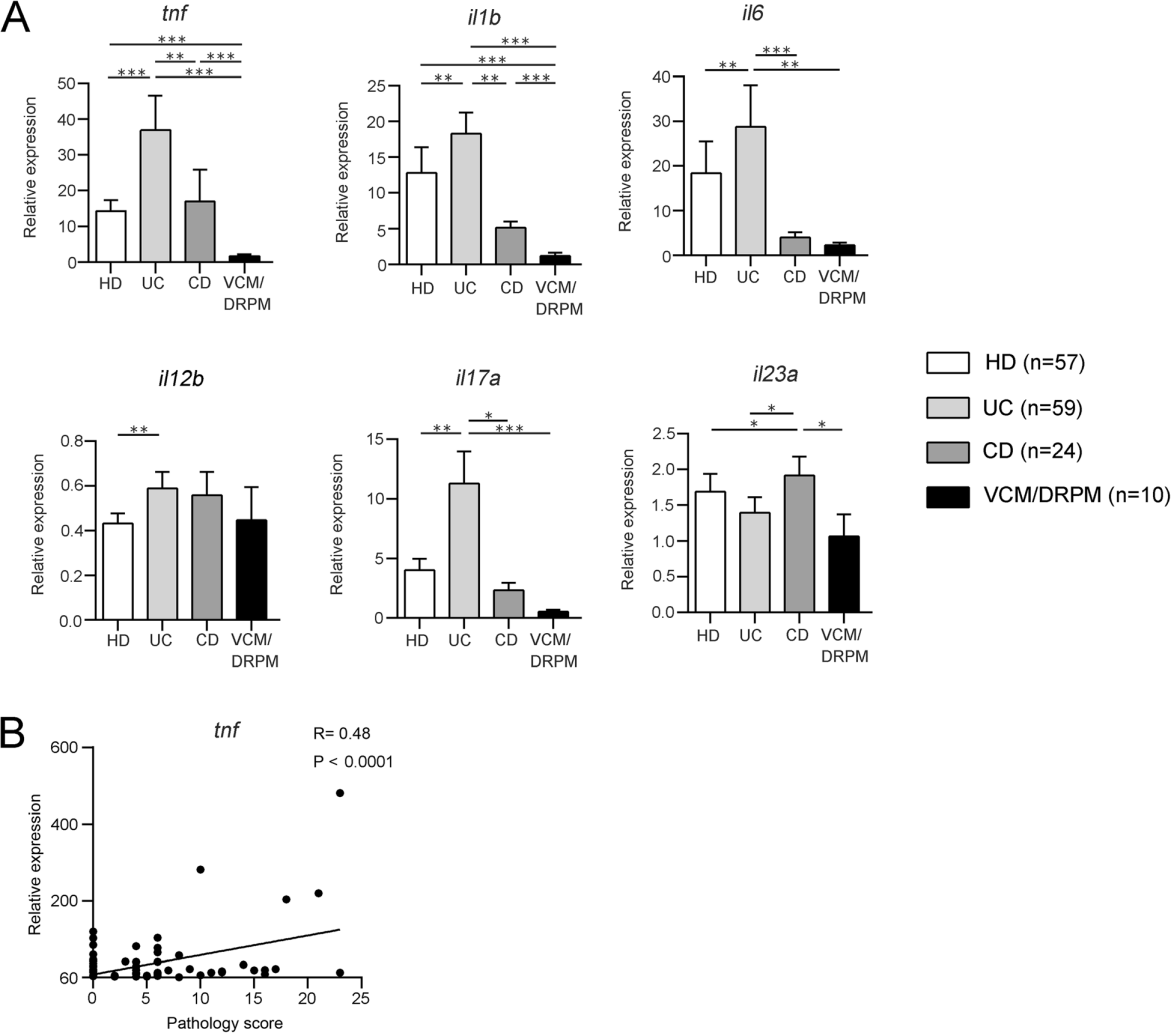
The fecal microbiota from IBD patients induce the expression of inflammatory cytokines in the colon. **a** On day 28 post-fecal transplantation, the mice were euthanized and mRNA expression in the colon was analyzed by real-time PCR. Values are the mean ± SE. Statistical differences between two values were analyzed using a Mann-Whitney *U* test. *P < 0.05; **P < 0.01; ***P < 0.001. **b** Linear regression line determined from plots showing changes in body weight (%) on day 28 and relative expression of *Tnf* in the murine colon. The regression coefficient (*R*) and *P* value are shown

Because expression of the inflammatory cytokines *Tnf* and *Il1b* in colon tissues was significantly higher in mice transplanted with HD, UC, and CD microbiota than in microbiota-depleted control mice (Fig. 3a), those cytokines were considered to be induced in response to various bacterial species present in the HD feces as well as the IBD feces. Among the three groups, the UC microbiota induced the highest levels of expression of *Tnf* and *Il1b* (Fig. 3a). The relative expression of Tnf in the colon was positively correlated with pathology scores in the same tissue (Fig. 3b). In comparison, the expression of *Il6* and *Il17a* was significantly higher in the colon tissues of UC microbiota-transplanted mice than in the HD or CD microbiota-transplanted mice or in the microbiota-depleted controls. However, the colonic expression of *Il23a* was significantly higher in the CD microbiota-transplanted mice than in the HD or UC microbiota-transplanted mice or the microbiota-depleted controls (Fig. 3a). The pattern of cytokine expression varied in individual mice, dependent on the feces of the individual human donor (Additional file 2: Figure S3). Therefore, the gene expression profiles of cytokines in the colon appear to be dependent on the fecal microbiota of the subjects.

### The abundance of *Enterococcus* in the fecal microbiota of mice is associated with inflammation in the colon

**Fig. 4:**
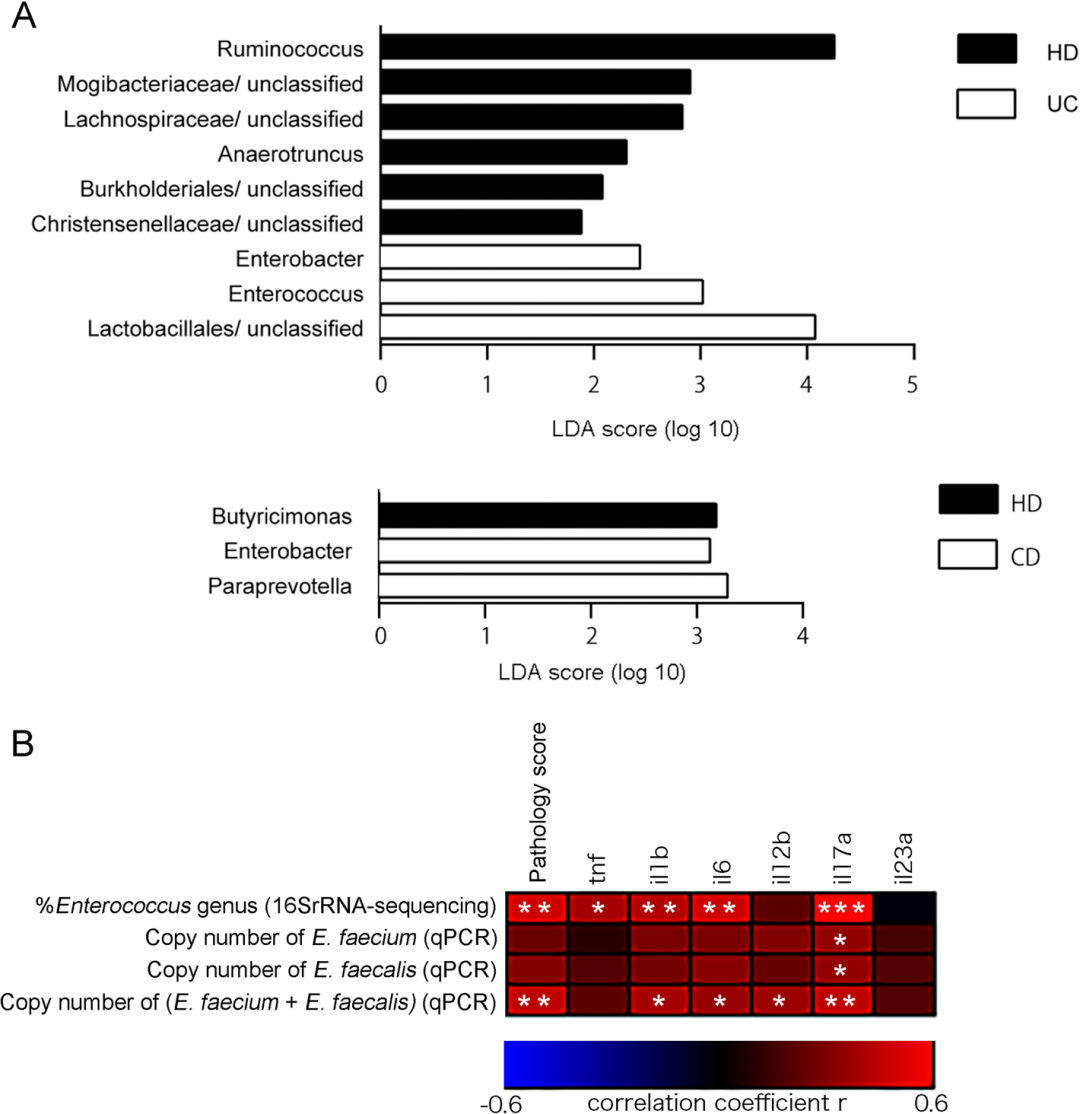
The abundance of *Enterococcus* in the fecal microbiota is associated with inflammation in the colon. The fecal transplantation experiment data shown in Figs. 2 and 3 were reanalyzed taking into consideration data generated from 16S rRNA-based metagenomic analysis of the mouse gut microbiota. **a** LDA was performed using LEfSe to determine significant differences in the relative abundance of specific genera in the feces of mice transplanted with HD and UC microbiota (top), or HD and CD microbiota (bottom). Differentially abundant genera for which the LDA scores indicated a *P* value < 0.05 are shown in the graphs. **b** Spearman’s rank correlation between pathology score or mRNA expression of certain cytokines in the colon tissues of the mice and the relative abundance or copy number of *Enterococcus* in the feces was visualized. Copy number of *E. faecium* or *E. faecalis* per gram of feces was determined by quantitative PCR. *P < 0.05; **P < 0.01; ***P < 0.001

Because the gut community composition of each of the human subjects was not completely reproduced in the transplanted mice, the characteristics of the gut microbiota of the transplanted mice were next examined by 16S rRNA-based metagenomic analysis. Significant differences in the abundance of nine genera were observed between the HD and the UC microbiota-transplanted mice, whereas only three genera were significantly altered between the HD and CD microbiota-treated mice (Fig. 4a). *Enterococcus* and *Enterobacter* were more abundant in the UC microbiota-transplanted gut communities compared with the HD group, while *Ruminococcus* and *Anaerotruncus* were less abundant in the UC group than in the HD microbiota-transplanted mice. *Paraprevotella* and *Enterobacter* were significantly more abundant in the CD microbiota-transplanted mice compared with the HD group. Because *E. faecium* was enriched in the microbiota of human UC patients compared with HD subjects (Fig. 1a), colonization of *E. faecium* in mouse colon was examined by culture of murine feces. On day 28 after fecal transplantation, colonization by *E. faecium* was detected significantly more in UC microbiota-transplanted mice than in HD microbiota-transplanted mice. No *E. faecium* was detected in any group before fecal transplantation (Additional file 2: Figure S4). Pathology and cytokine expression data from Figs. 2 and 3 were reanalyzed with respect to the proportion of *Enterococcus* in the microbiota of the mice determined by 16S rRNA-based metagenomic sequencing. Pathology scores and expression of *Tnf*, *Il1b*, *Il6*, and *Il17a* in the colon in mice positively correlated with the proportion of *Enterococcus* (Fig. 4b). However, when the copy number of *E. faecium* in mouse feces was determined by sensitive detection by quantitative PCR (Additional file 2: Figure S5a), the number of *E. faecium* only correlated with *Il17a* expression (Fig. 4b and Additional file 1: Table S6). Because *E. faecalis* is another major species that often colonizes clinical patients, the copy number of *E. faecalis* in mouse feces after fecal transplantation was also determined by PCR (Additional file 2: Figure S5b). Although the number of *E. faecalis* alone only correlated with *Il17a* expression, the sum of the number of *E. faecium* and *E. faecalis* positively correlated with pathology scores and expression of *Il1b*, *Il6*, *Il12b*, and *Il17a* (Fig. 4b and Additional file 1: Table S6). Thus, the presence of the genus *Enterococcus*, including *E. faecium* and *E. faecalis*, seemed to be correlated with colitis in mice after fecal transplantation.

### *E. faecium* causes colitis in *Il10*^−/−^ mice

**Fig. 5:**
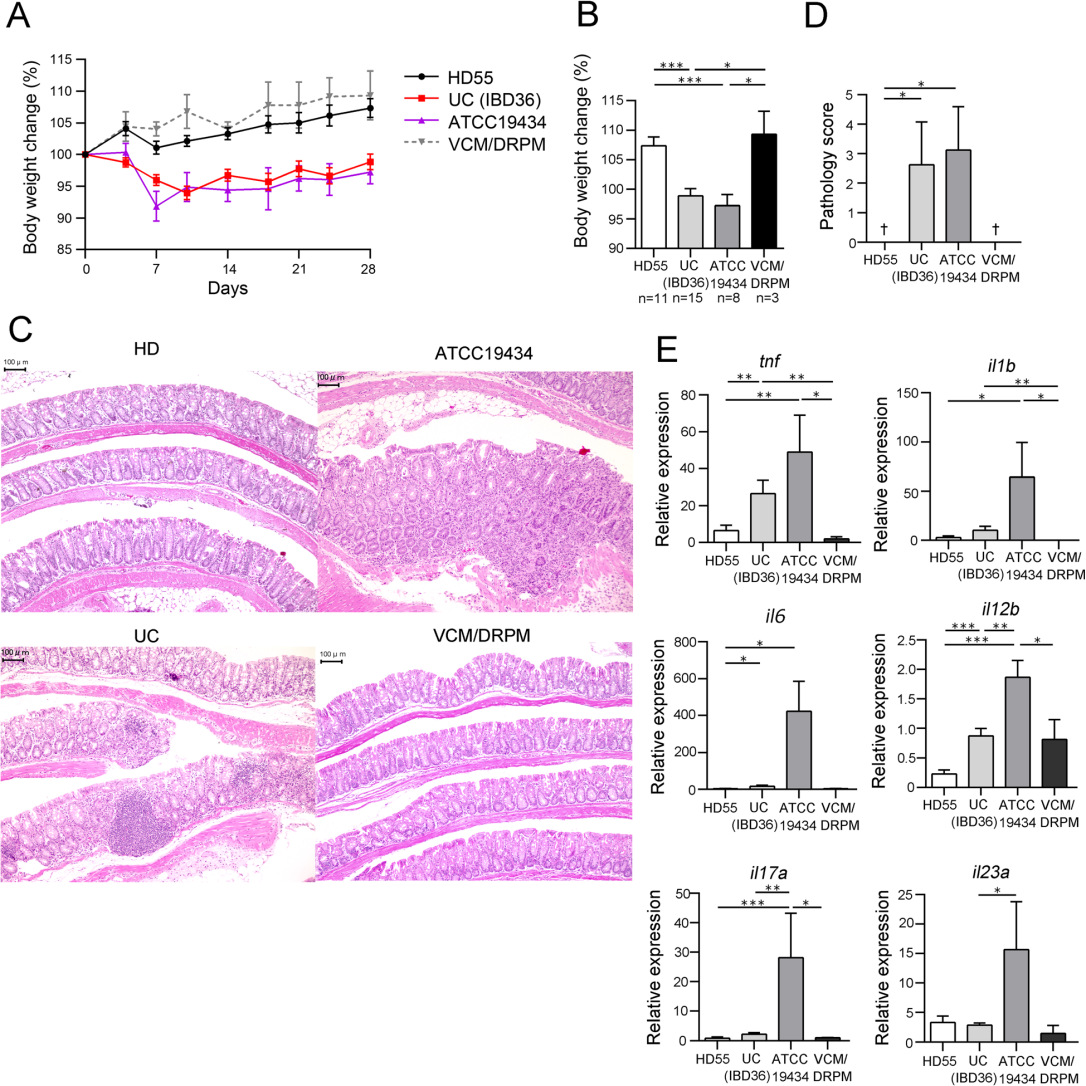
*E. faecium* in the gut causes colitis in *Il10*^−/−^ mice. Fecal transplantation from selected subjects (HD55 and IBD36) and inoculation of *E. faecium* strain ATCC 19434 was performed in microbiota-depleted *Il10*^−/−^ mice. The control group was treated with antibiotics (VCM/DRPM) in the absence of transplantation. **a** Changes in body weight (%) throughout the course of the experiment and **b** on day 28. **c** Representative histological sections of the murine colon at the time of euthanasia. Bars, 100 μm. **d** Mean pathology scores for each group of mice. †, average pathology score of 0. **e** mRNA expression levels of inflammatory cytokines in the colon as analyzed by real-time PCR. Values shown in **a**, **b**, **d**, and **e** are the mean ± SE. Statistical differences between two values were analyzed using a Mann-Whitney *U* test. *P < 0.05; **P < 0.01; ***P < 0.001

*E. faecalis* causes colitis in *Il10*^**−/−**^ mice according to a previous report [13], but a remaining question was whether *E. faecium* is causally involved in colitis. To further investigate the inflammatory effects of *E. faecium* in the colon, *E. faecium* strain ATCC 19434 was orally and rectally inoculated into the gut of microbiota-depleted *Il10*^−/−^ mice and colonization was confirmed (Additional file 2: Figure S6a and b). Fecal microbiota from selected subjects, HD subject 55 and UC patient IBD36 as negative and positive controls for inflammation, respectively, were also separately transplanted into microbiota-depleted mice. Increases in body weight were slower in the mice colonized with ATCC 19434 or IBD36 microbiota compared with mice transplanted with HD55 microbiota or the microbiota-depleted controls (Fig. 5a, b). Colon tissues of mice inoculated with ATCC 19434 or the IBD36 microbiota showed epithelial hyperplasia with lymphoplasmacytosis, obliteration of normal architecture, and erosion, particularly in the rectal segments, and mice belonging to these two groups had higher pathology scores compared with mice inoculated with HD55 microbiota and the microbiota-depleted controls (Fig. 5c, d). ATCC 19434 colonization significantly increased the expression of *Tnf*, *Il1b*, *Il6*, *Il17a*, and *Il12b* in colon tissues compared with HD55 microbiota transplantation. Therefore, we concluded that *E. faecium* ATCC 19434 is an inflammatory strain capable of promoting the expression of inflammatory cytokines and causing pathological changes in colon tissues.

### Subject-derived *E. faecium* strains induce different pathological changes and cytokine expression profiles in the colon

**Fig. 6:**
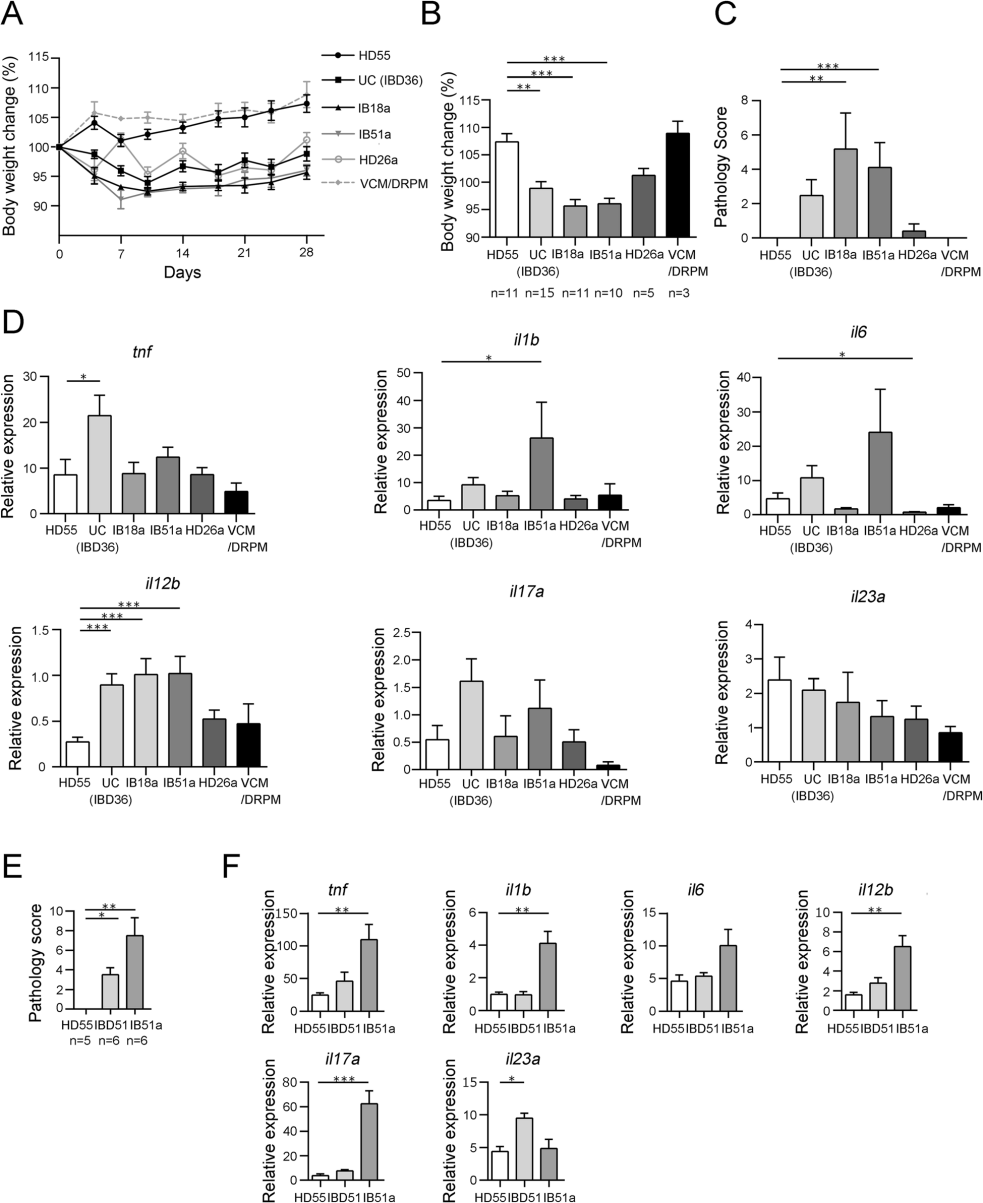
Subject-derived *E. faecium* strains lead to different pathology and cytokine expression profiles in the colon. Fecal suspensions from selected subjects (HD55 and IBD36) and *E. faecium* strain IB18a, IB51a, or HD26a suspensions were transplanted or inoculated into microbiota-depleted *Il10*^−/−^ mice. The control group was treated with antibiotics (VCM/DRPM) in the absence of transplantation. **a** Changes in body weight (%) throughout the course of the experiment and **b** on day 28. **c** Mean pathology scores of mice from each treatment group. **d** mRNA expression levels of inflammatory cytokines in the colon as analyzed by real-time PCR. **e**, **f** Suspension of HD55 or IBD51 feces or strain IB51a was gavaged into germ-free *Il10*^−/−^ mice. **e** Mean pathology scores of mice from each treatment group. **f** mRNA expression levels of inflammatory cytokines in the colon as analyzed by real-time PCR. Values shown in **a**–**f** are the mean ± SE. Statistical differences between a value and the HD55 control were analyzed using the Kruskal-Wallis test followed by Dunn’s test. *P < 0.05; **P < 0.01; ***P < 0.001

To examine whether *E. faecium* strains isolated from different subjects could promote colitis, multiple strains were isolated from the fecal samples. Colonization of strains in mice was confirmed by culture (Additional file 2: Figure S6c to f). Increases in the body weight of *Il10*^−/−^ mice inoculated with the UC microbiota-derived *E. faecium* strains IB18a or IB51a were slower than those recorded for HD55 microbiota-transplanted mice (Fig. 6a, b). In accord with body weight change, strain HD26a only caused modest pathological changes in the colon, whereas inoculation of IB18a or IB51a resulted in higher pathology scores in the colon compared with HD55 microbiota transplantation (Fig. 6c).

Consistent with these findings, strain HD26a did not increase the expression of inflammatory cytokines in colon tissues compared with HD55 microbiota transplantation (Fig. 6d). Interestingly, colon tissue cytokine expression profiles differed following inoculation with IB18a or IB51a. IB51a induced higher levels of expression of *Il1b* and *Il12b* compared with the HD55 microbiota, while IB18a only induced higher expression of *Il12b* (Fig. 6d). Commercially available *E. faecium* strain SF68, which is used as a probiotic drug for animals [14], was also tested by inoculation into microbiota-depleted *Il10*^−/−^ mice. SF68 did not cause pathological inflammation and increases of colonic cytokine expression, whereas UC-derived strain IB44a caused pathological colitis and increased expression of *Tnf*, *Il12b*, and *Il17a* (Additional file 2: Figure S7). Next, UC-derived strain IB51a was inoculated into germ-free *Il10*^−/−^ mice to see whether a single strain was sufficient to induce colitis. Inoculation with IB51a induced colonization with 100 times more *E. faecium* than transplantation of IBD51 feces that was the origin of IB51a (Additional file 2: Figure S6 g). Both monocolonization with IB51a and transplantation of IBD51 feces into germ-free mice induced more severe colitis than transplantation of HD55 feces (Fig. 6e). Strain IB51a increased expression of *Tnf*, *Il1b*, *Il12b*, and *Il17a* in the colon tissue, but IBD51 feces only increased expression of *Il23a* compared with HD55 feces (Fig. 6f). The cellular source of IL-17 in colonic lamina propria was both Th17 cells and CD3-negative cells (Additional file 2: Figure S8a and b). TNF and IL-6 were mainly produced by CD11c^−^MHC-class-II^+^ cells as well as smaller proportion of CD11c^+^MHC-class-II^+^ cells (Additional file 2: Figure S8c to f).

**Fig. 7:**
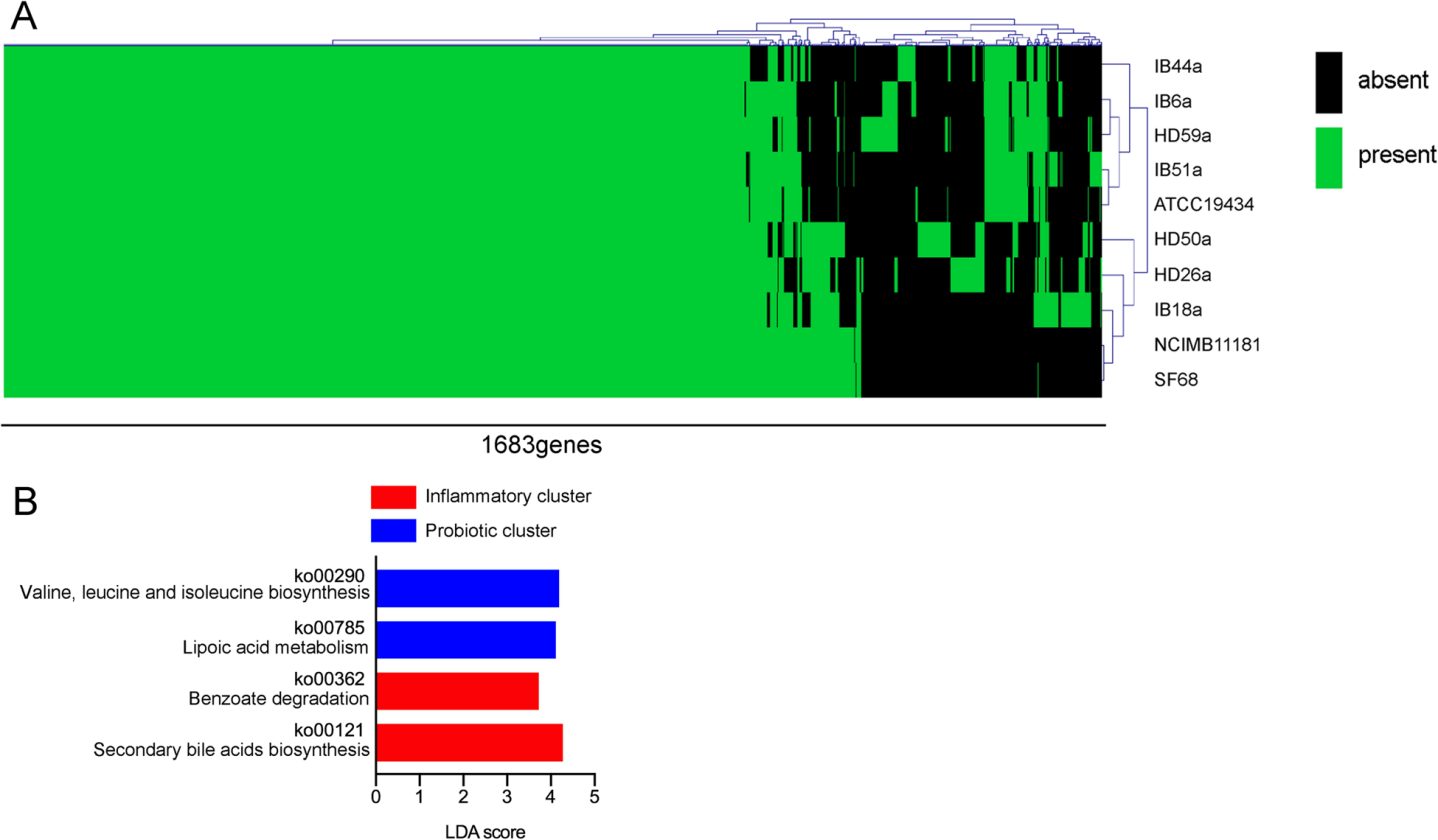
Genome analysis of 10 *E. faecium* strains reveals inflammatory and probiotic clusters. **a** Three (HD26a, HD50a, and HD59a) and 4 (IB6a, IB18a, IB44a, and IB50a) *E. faecium* strains were isolated from the feces of HD subjects and UC patients, respectively. The genotypes of 10 *E. faecium* strains, including the 3 HD-derived and 4 UC-derived strains, inflammatory strain ATCC 19434, and probiotic strains NCIMB 11181 and SF68, were examined by sequencing. All 1683 identified genes (except for those coding for hypothetical proteins) were used for hierarchical clustering analysis of the 10 *E. faecium* strains. **b** LDA was performed using LEfSe to identify significant differences in KEGG-based metabolic pathways in the genomes of the 10 strains to compare between the inflammatory cluster in which ATCC 19434 was included and the probiotic cluster in which NCIMB 11181 and SF68 were included. Differentially abundant pathways for which the corresponding LDA scores indicate *P* < 0.05 are shown in the graphs

To assess whether the different pathology and cytokine expression profiles in the colon tissues were caused by differences in the genotypes of the various *E. faecium* strains, genomic DNA from each of the strains was sequenced. In total, 1683 genes (not counting genes coding for hypothetical proteins) were annotated from the DNA sequence reads derived from 10 *E. faecium* strains. Hierarchical clustering analysis of the 1683 genes generated 2 apparent clusters among the 10 strains (see Fig. 7a and the gene list described in Additional file 3: Figure S9). *E. faecium* strains NCIMB 11181 and SF68, both of which have previously been used as probiotics in animals [14, 15], were closely clustered, but pro-inflammatory *E. faecium* strain ATCC 19434 was in the other cluster. Three strains derived from the feces of UC patients, IB51a, IB6a, and IB44a, were clustered with pro-inflammatory strain ATCC 19434, while two strains derived from the feces of HD subjects, HD26a and HD50a, were clustered with probiotic strains NCIMB 11181 and SF68, although HD-derived strain HD59a was grouped into the other cluster. IB18a, which induced a different cytokine expression profile from that of strain IB51a, was distant in the plot from IB51a and was much closer to the probiotic strains (Fig. 7a and Additional file 3: Figure S9). KEGG-based analysis was used to identify metabolic pathways present in the probiotic cluster and the inflammatory cluster. The *E. faecium* strains in the inflammatory cluster possessed more or less abundant pathways of secondary bile acid biosynthesis or lipoic acid metabolism, respectively, compared with those in the probiotic cluster (Fig. 7b). Activity of bile salt hydrolase (BSH) which is involved in the first process of secondary bile acid biosynthesis was dependent on type of strains, and HD26a and HD50a, two strains derived from HD feces, lacked the BSH activity (Additional file 4: Figure S10a). Because lipoic acids are anti-oxidants according to previous reports [16], reactive oxygen species (ROS) level in *E. faecium* culture was also measured. Relative ROS level in culture supernatant of the strains in the inflammatory cluster was higher than the probiotic cluster (Additional file 4: Figure S10b and c). Taken together, these results suggest that the *E. faecium* strains derived from the subjects’ microbiota were genetically diverse and that this genetic diversity might be associated with the pathological diversity of the strains in the colon.

### Presence of *E. faecium* in the gut is associated with disease extent and the requirement for combination therapy in UC patients

**Fig. 8:**
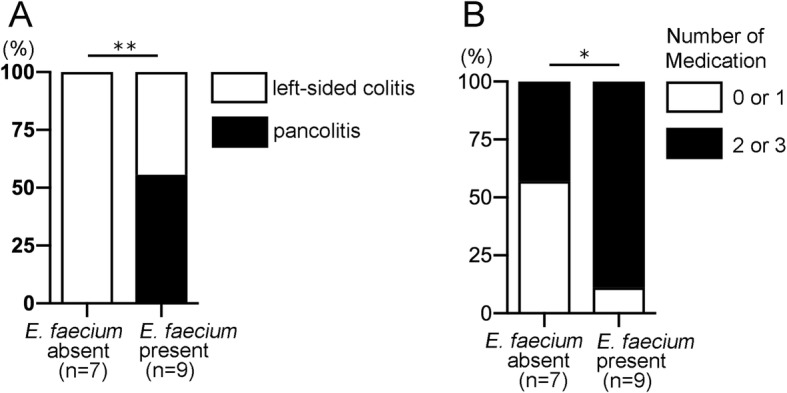
The presence of *E. faecium* is associated with disease extent and the requirement for combination therapy. The disease characteristics and treatment regimens of the 16 UC patients enrolled in this study were obtained from medical records and assessed to identify any association with the presence or absence of *E. faecium* in the gut microbiota as determined by PCR. **a** Proportions of UC patients with pancolitis or left-sided colitis are shown relative to the presence of *E. faecium* in the feces. **b** Proportions of UC patients treated with single or multiple mediations are shown relative to the presence of *E. faecium* in the feces. Medications included mesalazine, corticosteroids, azathioprine, mercaptopurine, tacrolimus, infliximab, and adalimumab. The number of subjects per category (*n*) is indicated. *P < 0.05 by chi-squared test

To investigate the clinical association between *E. faecium* colonization and UC, data from the 16 UC subjects were examined in more detail. Disease characteristics and treatment regimens were obtained from medical records and were assessed to identify any association with the presence or absence of *E. faecium* as determined from the PCR data. Fifteen UC patients received medication; the Mayo score, a disease activity score of UC, can be influenced by treatment. Thus, the presence of *E. faecium* was not associated with the Mayo score (Additional file 4: Figure S11). However, in terms of disease type, the prevalence of pancolitis was higher among UC patients colonized with *E. faecium* compared with those that were not colonized with *E. faecium* (pancolitis: *E. faecium*-negative, 0%; *E. faecium*-positive, 55.6%; *P* = 0.0087; Fig. 8a). In addition, *E. faecium*-positive UC patients tended to be treated with a greater number of mediations than UC patients without *E. faecium* in the colon (number of medications = 0 or 1, and 2 or 3: *E. faecium*-negative, 57.1% and 42.8%; *E. faecium*-positive, 11.1% and 88.8%; *P* = 0.048; Fig. 8b). Thus, the presence of *E. faecium* in the gut was associated with disease extent and the requirement for combination therapy in UC patients.

## Discussion

A causal link between specific bacterial species and the pathogenesis of IBD remains controversial because of the multifactorial causes of IBD and difficulties surrounding reproducing the disease in mouse models. In this study, we attempted to identify a causal relationship between specific bacterial species and IBD by conducting a metagenomic analysis of colitis-susceptible *Il10*^−/−^ mice transplanted with fecal microbiota from 37 human subjects. The fecal microbiota from both UC and CD patients caused pathological inflammation in the colon tissues of experimental animals, while microbiota from HD rarely caused severe colitis (Fig. 2c–e). The microbiota from UC patients induced expression of the *Il6–Il17a* axis in the colon, whereas the microbiota from CD patients induced expression of *Il23a* (Fig. 3a). *Enterococcus* was differentially abundant in the microbiota of UC patients compared with the HD group (Fig. 1a), which was replicated in the mice transplanted with the UC microbiota (Fig. 4a and Additional file 2: Figure S5). *E. faecium* strain ATCC 19434 (Fig. 5c–e), along with strains isolated from UC patients (Fig. 6c, d), caused pathological inflammation and upregulation of cytokine expression in the colon. The genotypes of 10 analyzed *E. faecium* strains were different and could be separated into two major clusters: one containing two probiotic strains and the other containing pro-inflammatory strain ATCC 19434 (Fig. 7a). Thus, these findings implicate *E. faecium* strains with a particular genotype in colonic inflammation in genetically susceptible hosts.

The metagenomic analysis carried out in this study revealed obvious differences in the composition of the microbiota from UC or CD patients compared with that from HD (Fig. 1c). Several of the observed differences between the IBD patients and HD were consistent with previous reports, such as the decreased abundance of *F. prausnitzii* [7, 17] and *Eubacterium rectale* [17] in the microbiota of UC patients, and the increased abundance of *Escherichia* species, including *Escherichia coli* [3], in the CD microbiota. According to previous reports, enterococci are more abundant in the fecal matter [5, 18] and mucosa [19] of CD patients compared with healthy controls. In this analysis, *E. faecium* was more abundant in the UC microbiota, but not the CD microbiota, compared with HD (Fig. 1a). Although the results of metagenomic analyses are often affected by large interindividual differences arising from differences in factors such as geographic location, diet, and antibiotic use, the overall results of the compositional difference between HD and IBD in the current study are fairly consistent with previous reports. Because most of the patients enrolled in this study were being treated for their disorder, it is unclear whether the observed changes in microbiota are associated with the disease onset. Longitudinal metagenomic analysis from a naïve state to a treated state for IBD patients is therefore necessary to clarify the association between disease onset and specific bacterial species.

Inflammatory cytokines are crucial to the pathogenesis of IBD [20]. TNF is a central driver of inflammation in mucosal tissues, as evidenced by the anti-inflammatory effects of a neutralizing antibody against TNF that is currently used worldwide for the treatment of both UC and CD [21, 22]. IL-6 produced by lamina propria myeloid cells prevents apoptosis of T cells and activates macrophages [23]. Antibody-mediated blockade of IL-6 signaling resulted in clinically beneficial responses in a subgroup of CD patients in a clinical trial [24]. IL-6 is also necessary for differentiation of Th17 cells. IL-17A produced by Th17 cells in the lamina propria is abundant in the mucosa of both UC and CD patients [20]. Although neutralization of IL-17A was not effective in the relief of colitis in a mouse model [25] or in a clinical study of CD patients [26], another Th17-type cytokine, IL-21, also has pro-inflammatory effects and may be a suitable therapeutic target [20]. IL-23 is mainly produced by antigen-presenting cells to stabilize Th17 cells, but also activates macrophages. A neutralizing antibody against IL-12p40, a subunit of IL-23, is now used for the treatment of CD patients worldwide [27]. Interestingly, in this study, cytokine expression profiles in colon tissues of *Il10*^−/−^ mice were dependent on the composition of the transplanted microbiota. The UC microbiota tended to increase the expression of the *Il6–Il17a* axis, while the CD microbiota increased *Il23a* expression in the colon (Fig. 3a). In addition, cytokine profiles were dependent on the genotypes of the *E. faecium* strains in the *Il10*^−/−^ mice (Fig. 6d). Strain IB51a increased expression of *Tnf*, *Il1b*, *Il12b*, and *Il17a* in gnotobiotic conditions, but original IBD51 feces only increased *Il23a* expression (Fig. 6f). Even in the same host background, differences in the bacterial community composition might affect the specific cytokine expression profiles of the colon tissues. This study also showed distinct differences in bacterial species composition between the UC and CD microbiota and the HD microbiota (Fig. 1a, b), as previously reported [3]. Similar to the current study, differences in *Klebsiella pneumoniae* genotype induced different inflammation profiles in the colon of *Il10*^−/−^ mice [28]. Therefore, accumulating information regarding the relationship between the expression of intestinal cytokines and the composition of the gut microbiota, as well as the genetic background of the host, may help in the development of individualized treatment protocols to appropriately neutralize various cytokines.

*Il10*^−/−^ mice, which lack anti-inflammatory cytokine IL-10 and show spontaneous colitis in a gut microbiota-dependent manner [12], were used in this study. Because *IL10* gene polymorphisms contribute to UC [29] and CD [30] susceptibility in humans, the *Il10*^−/−^ mouse model is a good approximation of the genetic susceptibility of IBD patients. However, multiple alleles are usually involved in the pathogenesis of human IBD [31], and not all IBD patients have an *IL10* polymorphism. Therefore, further investigation using other mouse models of IBD is necessary to validate the results of the current study, although mouse models of genetic susceptibility to colitis are scarce. Microbiota-dependent patterns of cytokine production also need to be confirmed in other mouse models. A limitation of this study was that transplantation of human feces into mice after depletion of native mouse microbiota with antibiotics resulted in incomplete restoration of the human microbiota (Additional file 2: Figure S1). Thus, we were able to study effects of subject-unique microbiota by fecal transplantation, but the microbiota were not the same as the original microbiota of the donor human. Despite this limitation of the mouse experiments in this study, the presence of *E. faecium* was associated with increased disease extent and the requirement for multiple medications in UC patients with a non-specific genetic background. Taken together, the results of the current study suggest that *E. faecium* may promote colonic inflammation in UC patients. To validate the inflammatory effects of *E. faecium* in IBD patients, well-designed clinical studies using a larger UC population are necessary.

Probiotic *E. faecium* strains NCIMB 11181 [15] and SF68 [14, 32] have been used for many years to maintain animal health; however, the detailed molecular mechanisms underlying their probiotic effects have not been fully elucidated. In comparison, the virulence factors of *E. faecium* have been extensively studied because of the significant health concern caused by vancomycin-resistant *E. faecium* strains in hospitalized patients [33]. Unlike streptococci and staphylococci, most enterococci do not produce pro-inflammatory toxins; instead, they possess many genes encoding adhesion proteins that mediate adherence to host tissues [33]. Acm is one such adhesion protein produced by clinical *E. faecium* strains. Acm binds collagen in host tissues, and deletion of *acm* results in attenuation of *E. faecium*-mediated endocarditis in an animal model of disease [34]. Surface protein Esp in *E. faecium* appears to have been acquired as part of a pathogenicity island, and promotes biofilm formation and urinary tract infection in mice [35]. Adhesion and biofilm formation are important properties involved in the translocation of luminal bacteria in colon tissues [33]. The genetic analysis of 10 *E. faecium* strains in the current study identified that pathways for secondary bile acid biosynthesis were abundant in the pro-inflammatory cluster of *E. faecium* strains. Because particular types of bile acids control biofilm formation of *Enterococcus* [36], polarization of host macrophages, generation of oxidative stress [37], and expansion of pathobionts in the colon [38], the ability of “inflammatory” *E. faecium* to alter the composition of bile acids in the intestinal environment may be a key feature of pathogenesis of *E. faecium* in colitis. Abundance of the pathway of lipoic acid metabolism was also different between the inflammatory and the probiotic clusters. Lipoic acids are considered as anti-oxidants and effective to relieve colitis in pre-clinical animal studies [16]. Because *E. faecium* strains in the inflammatory cluster possessed ability to generate more ROS than the probiotic cluster, ROS-related pathogenic effect may be involved in the colitis induced by inflammatory *E. faecium*. In several fecal transplantation, *E. faecium* caused colitis in *Il10*^−/−^ mice with low bacterial load (Additional file 2: Figure S5). Ability of inflammatory *E. faecium* in generating metabolites related to bile acids and ROS might account for a reason why low bacterial load of the strains promoted colitis.

## Conclusions

Metagenomic analysis of the fecal microbiota of IBD patients and transplantation of feces from the corresponding patients into genetically susceptible animals confirmed a causal relationship between inflammatory *E. faecium* strains and colitis. Because the cytokine expression profile in the colon may partly be dependent on the gut microbiome, characterizing the composition of a patient’s gut microbiota may lead to personalized antibody therapy for IBD patients in the future.

## Methods

### Clinical study population

This study aimed to examine fecal microbiota of IBD patients who visited Kanazawa University Hospital in various stages including both active and remission stages. Between February 2014 and March 2015, 60 IBD patients enrolled in the study. Among them, 55 subjects received colonoscopy to evaluate disease status. To investigate the composition and metagenomes of the gut microbiota of the different patient groups, fecal samples were collected from patients immediately after enrollment and stored at − 80 °C. Among the 55 subjects, 16 UC patients and 8 CD patients provided us with good and sufficient feces immediately after enrollment, and these feces were used for the study. Feces that were too watery or that were left at room temperature for > 3 h were not used. Patients received both written and oral information before consenting to participate in this study. Healthy individuals were first tested to confirm that they met the following inclusion criteria: body mass index < 25 kg/m^2^, normal blood pressure, normal serum cholesterol, normal blood glucose and hemoglobin A1c levels, normal serum aspartate transaminase and alanine aminotransferase levels, no anemia, no fatty liver noted by ultrasonography, and no past history of cancer. In total, 13 individuals were enrolled as HD. Immediately after consent, feces were collected from the HD and stored at − 80 °C. This study was approved by the Ethics Committee of Kanazawa University (approval number 2012-109).

### Animals

Male and female *Il10*^−/−^ mice with a C57BL/6j background were purchased from Jackson Laboratories (Bar Harbor, ME, USA). Mice were housed in individually ventilated racks in an animal facility with access to autoclaved food and water ad libitum. Stringent husbandry techniques, including a strictly enforced order of cage handling and scrupulous attention to environmental sanitization, were followed to prevent contamination. Germ-free *Il10*^−/−^ mice with a C57BL/6j background were generated by Japan CLEA (Tokyo, Japan). Germ-free or gnotobiotic mice were housed in germ-free isolators (ICM, Tsukuba, Japan) in the animal facility of Kanazawa University. All animal procedures were performed in accordance with the Guidelines on the Care and Use of Laboratory Animals issued by Kanazawa University and were approved by the Ethical Committee for Animal Experiments of Kanazawa University (approval number 143267).

### Fecal transplantation and bacterial inoculation

Frozen fecal samples were thawed and resuspended in a 10 times volume (w/v) of reduced phosphate-buffered saline (PBS) containing 0.1% resazurin (w/v) and 0.05% l-cysteine-HCl (Sigma-Aldrich, St. Louis, MO, USA) under anaerobic conditions (80% N_2_, 10% H_2_, 10% CO_2_) in an anaerobic chamber (RUSKINN, Bridgend, UK), before being filtered through a 100-μm pore size nylon membrane filter. Depletion of the gut microbiota was performed as previously described [39], with slight modification. Briefly, 8- to 12-week-old C57BL6 mice were treated with antibiotics, including doripenem hydrate (0.25 g/L; Shionogi, Japan) and vancomycin hydrochloride (0.5 g/L; Shionogi), via water for 2 weeks prior to experimentation to deplete the gut microbiota. Fecal suspensions were then inoculated into the microbiota-depleted mice by oral gavage (100 μL) and rectally (100 μL) using a flexible plastic tube on days 1 and 2 after cessation of antibiotic treatment. For bacterial inoculation experiments, *E. faecium* strains ATCC 19434 (ATCC, Manassas, VA, USA), NCIMB 11181 (NCIMB, Aberdeen, UK), SF68 (also known as NCIMB 10415; PURINA, Largo, FL, USA), or human feces-derived strains were prepared at a concentration of 2.5 × 10^8^ colony-forming units (CFU)/100 μL in PBS. Suspensions were then inoculated into microbiota-depleted mice as described for the inoculation of fecal suspension. The microbiota-depleted mice were housed individually in separate isolators following inoculation with fecal suspension or specific bacterial strains.

### Whole-genome shotgun and 16S rRNA amplicon sequencing analysis of fecal and bacterial DNA extracts

Microbial DNA was extracted from the stored fecal samples using a PowerFecal DNA Isolation Kit according to the manufacturer’s instructions (MO BIO, Carlsbad, CA, USA), although 0.1-mm glass beads (MO BIO) rather than 0.7-mm garnet beads were used to homogenize feces.

For whole-genome shotgun sequencing, DNA fragmentation was performed using a Nextera DNA Library Prep Kit (Illumina, San Diego, CA, USA) with incubation at 55 °C for 5 min. Following product clean-up using a DNA Clean & Concentrator-5 Kit (ZYMO, Irvine, CA, USA), indexing PCR was performed using a Nextera Index Kit (Illumina), with an initial cycle of 72 °C for 3 min and 98 °C for 30 s, followed by 5 cycles of 98 °C for 10 s, 63 °C for 30 s, and 72 °C for 3 min. PCR products were purified from oligo DNA contaminants using AMPure XP beads (Beckman Coulter, Fullerton, CA, USA) and then quantified using a 2100 Bioanalyzer and High Sensitivity DNA Kit (Agilent Technologies, Santa Clara, CA, USA). The libraries were pooled, and sequencing was performed using the MiSeq system (Illumina) with a MiSeq Reagent Kit V3 (600 cycles; Illumina).

Preprocessing of the acquired sequences was performed as follows. After trimming of bases with low-quality scores from the ends of the acquired sequences, the sequences were filtered at a Q-score cutoff of 20 using FASTX Toolkit [40]. Paired-end joining was performed using MacQIIME v1.9.1 [41]. After mapping of the resultant sequences against UCSC human reference genome hg19 using bowtie2 version 2.2.4 [42], human genome sequences were removed using SAMtools-1.2 [43]. Finally, PCR duplicates were removed using PRINSEQ version 0.20.4 [40]. The average number of reads per sample before and after quality control was 1,544,241 ± 687,438 and 1,106,822 ± 560,821 (mean ± SD), respectively.

The resultant FASTA files were used for taxonomic analysis of the fecal samples using MetaPhlAn2 version 2.0.0 [44]. Metabolic pathways indicated by the sequences were identified using HUMAnN2 [45] version 0.1.9. A KEGG version 56 database was generated using DIAMOND version 0.7.5 [46] and used for HUMAnN2 analysis. After normalization of the abundance to 1 million reads, comparisons between groups were performed by LDA using the linear discriminative analysis effect size (LEfSe) tool [47]. The Bray-Curtis dissimilarities among the gut microbiota communities were calculated based on taxonomic or metabolic pathway data, and the resultant distances were visualized by PCoA. Statistical differences between communities were tested using PERMANOVA with MacQIIME version 1.9.1.

Whole-genome shotgun sequencing of individual *E. faecium* strains was performed as described for fecal metagenome analysis. Assembly of the obtained paired-end FASTQ files was performed using SPAdes version 3.12.0 [48]. The resultant scaffold FASTA files were used to annotate genomic features in PROKKA version 1.12 [49]. Hierarchical clustering of 1683 genes from the 10 *E. faecium* strains annotated by PROKKA was performed using an average linkage method in Genesis version 1.7.6 [50]. KEGG-based metabolic pathways indicated by the sequences were also identified using HUMAnN2 version 0.1.9, and comparisons between “inflammatory” and “probiotic” clusters were performed by LEfSe.

For 16S rRNA gene sequencing analysis, PCR amplicons were prepared using primers targeting the V3–V4 region of the gene, with Illumina adapter sequences:

Forward 5′-TCGTCGGCAGCGTCAGATGTGTATAAGAGACAGCCTACGGGNGGCWGCAG-3′; Reverse 5′-GTCTCGTGGGCTCGGAGATGTGTATAAGAGACAGGACTACHVGGGTATCTAATCC-3′.

PCR reactions were performed using KAPA HiFi HotStart Ready Mix (KAPA Biosystems, Wilmington, MA, USA) with an initial step at 95 °C for 3 min, followed by 25 cycles of 95 °C for 30 s, 55 °C for 30 s, and 72 °C for 30 s, with a final elongation at 72 °C for 5 min. Following purification, indexing PCR was performed using a Nextera XT Index Kit (Illumina) with an initial step at 95 °C for 3 min, followed by 8 cycles of 95 °C for 30 s, 55 °C for 30 s, and 72 °C for 30 s, with a final elongation step at 72 °C for 5 min. Libraries were pooled and mixed with the PhiX Control Library (Illumina) before being sequenced using the MiSeq system with a MiSeq Reagent Kit V3 (600 cycles). Downstream processing of the sequences was performed using MacQIIME version 1.9.1 [41]. After paired-end sequence joining and trimming of low-quality reads, the resulting sequences had lengths 442–464 bp. The average number of reads per sample after quality control was 96,311 ± 35,047 (mean ± SD). Operational taxonomic units (OTUs) were assigned using UCLUST. For OTU analysis, sequences were clustered, and then, those with > 97% similarity were binned into the same OTU. Taxonomic assignment of representative sequences from each OTU was performed using RDP Classifier using the Greengenes reference database [51] clustered at 97% identity. Genus-level taxonomy was summarized and used for subsequent analyses. Following the generation of a phylogenetic tree, unweighted UniFrac distances for the gut microbiota communities were calculated, with the resultant distances visualized by PCoA.

All sequencing data and metadata were archived in the NCBI Sequence Read Archive under BioProject numbers PRJNA511372 and 511382.

### Real-time PCR

Total RNA extraction and cDNA synthesis were performed as previously described [52]. Briefly, colon tissue samples were fixed in RNAlater RNA Stabilization Reagent (Qiagen, Hilden, Germany) and stored at − 80 °C until processing. Total RNA was isolated from the specimens using an RNeasy Mini Kit (Qiagen) according to the manufacturer’s instructions. A High Capacity cDNA Archive Kit (Applied Biosystems, Foster City, CA, USA) was used to reverse transcribe 500 ng of total RNA into first-strand cDNA. Quantitative PCR was carried out using a real-time 7900HT Sequence Detection System (Applied Biosystems). TaqMan probes used for real-time PCR were as follows: *Tnf* (Mm00443260_g1), *Il1b* (Mm00434228_m1), *Il6* (Mm00446190_m1), *Il12b* (Mm01288989_m1), *Il17a* (Mm00439618_m1), and *Il23a* (Mm00518984_m1) (Applied Biosystems). To control for variations in the amount of DNA available for PCR, target gene expression in each sample was normalized relative to the expression of endogenous control gene *β-actin* (Applied Biosystems) using the ΔΔCt method. PCR amplification was performed with an initial cycle of 50 °C for 2 min and 95 °C for 10 min, followed by 40 cycles of 95 °C for 15 s and 60 °C for 1 min.

Detection of *E. faecium* and *E. faecalis* in fecal samples was performed as previously described [53]. Briefly, 2 ng of fecal DNA was amplified with SYBR Green (Qiagen) and primers Efm12 or Efl6 [53] to detect *E. faecium* or *E. faecalis*, respectively, by reaction at 94 °C for 10 min, followed by 40 cycles of 94 °C for 15 s and 60 °C for 1 min. To draw a standard curve, DNA extracted from *E. faecium* strain ATCC 19434 or *E. faecalis* strain V583 (ATCC) was used, and copy number was calculated.

### Histological analysis

Colon samples were fixed in 10% buffered formalin using a “Swiss roll” technique [54]. Fixed tissues were embedded in paraffin and stained with hematoxylin and eosin. Hematoxylin and eosin-stained sections were used for histological scoring. Tissue sections were coded to eliminate pathologist bias and scored as previously described [54], with slight modification. Briefly, the proximal colon, distal colon, and rectum from each mouse were scored based on the severity of mucosal epithelial changes, degree of inflammation, and extent of pathology (Additional file 1: Table S7). The segment score was calculated by summing the severity scores: [segment score = mucosal score + inflammation score + extent of segment affected in any manner (extent 1) + extent of segment affected at level 3 or 4 in M or I score (extent 2)], where the maximum segment score was 15. The total score for each mouse was calculated by summing the scores from the individual segments (maximum total score of 45). Subsequently, the mean total scores were calculated for each group.

### Bacterial culture

*E. faecium* strains were proliferated in Brain Heart Infusion (BHI) medium (Eiken Chemical Co., Tokyo, Japan). To isolate *E. faecium* strains from human feces, fecal samples were streaked onto ECS agar plates (Eiken Chemical Co.). Single colonies were picked from the selection agar and cultivated on EF agar plates (Nissui, Japan), upon which *E. faecalis* forms purple colonies and *E. faecium* produces orange colonies. Individual orange colonies were picked and proliferated in BHI broth. Species identification was carried out using PCR-based methods as previously described [53, 55]. Finally, *Enterococcus* species were confirmed by whole-genome shotgun sequencing as described in the “Whole-genome shotgun and 16S rRNA amplicon sequencing analysis of fecal and bacterial DNA extracts”section. Fecal suspension was cultured on EF agar plates to obtain CFU counts, and colonies were confirmed as *E. faecium* by PCR-based methods.

### Intracellular cytokine staining by flow cytometry

Single cell suspension was obtained from the colonic lamina propria. The colon tissues were cut into 1-cm pieces and incubated with 5 mM ethylenediaminetetraacetic acid (Invitrogen) in Hanks’ balanced salt solution at 37 °C for 20 min, followed by washing with HBSS three times. Next, the tissue was minced with scissors and transferred into conical tubes containing 10 mL RPMI 1640 medium (Thermo Fisher, Waltham, MA, USA) and 0.5 mg/mL collagenase (Sigma-Aldrich, St. Louis, MO, USA) and incubated at 150 rpm and 37 °C for 30 min. The single cell suspension was collected and passed through 100-μm and 40-μm cell strainers and washed twice with RPMI 1640. For analysis of IL-17A expression, the single colonic lamina propria cells were restimulated in complete RPMI 1640 with 5 ng/mL phorbol 12-myristate 13-acetate and 500 ng/mL ionomycin in the presence of 2 μM monensin (Sigma-Aldrich) for 3 h at 37 °C. For analysis of IL-6 and TNF expression, the cells were incubated in complete RPMI 1640 in the presence of 2 μM monensin for 3 h at 37 °C. Dead cells were excluded from all analyses using a LIVE/DEAD Fixable Aqua Dead Cell Stain Kit (Thermo Fisher). For intracellular cytokine staining, cells were fixed with IC Fixation Buffer (Thermo Fisher). Fluorescent dye-conjugated antibodies were used to stain CD45, CD3 (BD Biosciences, Franklin Lakes, NJ, USA), CD4, CD11c, MHC-class II, IL-17, TNF, and IL-6 (BioLegend, San Diego, CA, USA). All data were acquired on a FACS Aria II flow cytometer (BD Biosciences) and analyzed using FlowJo V.10 software (FlowJo, Ashland, OR, USA).

### BSH assay

*E. faecium* strains were tested for hydrolase activity against glyco-conjugated bile acid as previously described [56]. Overnight MRS broth cultures were streaked on MRS agar supplemented with 2 mM GDCA (Sigma; G3258). The plate was then incubated for 48 h at 37 °C. BSH activity was detectable when deoxycholic acid precipitated in the agar medium around a colony.

### ROS measurement in culture supernatant

OxiSelect In vitro ROS/RNS assay kit (green fluorescence) (Cell Biolabs, cat. STA-347) was used to measure the ROS/RNS (reactive nitrogen species) in culture supernatant as previously described [57] with modification. After *E. faecium* strains were cultured in BHI medium overnight, 50 μL of the supernatants were mixed with 50 μL of catalyst (provided in the kit) to accelerate the oxidative reaction. Following 5 min incubation at room temperature, 100 μL of DCFH-DiOxyQ probe solution was added to the mixture to measure the total free radical population. DCFH probe can react with free radical molecules that are representative of both ROS and RNS. The samples were incubated at room temperature for 30 min and read with a fluorescence plate reader at Ex/Em = 480/530 nm. The standard curve of H_2_O_2_ was used to semi-quantify the free radical content in the culture supernatant samples. Then, relative ROS level was determined.

### Statistical analyses

Statistical analyses of taxonomic and KEGG pathway comparisons between microbiota communities were performed using the LEfSe tool [47]. Statistical differences between two values were analyzed using a Mann-Whitney *U* test or the Kruskal-Wallis test followed by Dunn’s test for correction of multiple comparisons in GraphPad Prism 7 (GraphPad Software, San Diego, CA, USA). Statistical differences between treatments or in the percentages of disease extent were analyzed by *χ*^2^ test in GraphPad Prism 7. A *P* value < 0.05 was considered statistically significant. Linear regression coefficients between pathology scores, percentage body weight changes, and *Tnf* expression levels were calculated using GraphPad Prism 7. Spearman’s rank correlation was analyzed using GraphPad Prism 7.

## Supplementary information

**Additional file 1: Table S1.** Baseline demographic characteristics of the subjects. **Table S2.** Disease characteristics of the UC patients. **Table S3.** Disease characteristics of the CD patients. **Table S4.** Comparison of KEGG pathway abundance in fecal microbiota between HD and UC subjects by linear. **Table S5.** Comparison of KEGG pathway abundance in fecal microbiota between HD and CD subjects by linear discriminative analysis. **Table S6.** Correlation between abundance of fecal Enterococcus and pathology/cytokine production. **Table S7.** Scoring system for histological evaluation of mouse colon tissues.

**Additional file 2: Figure S1.** β-Diversity analysis of the bacterial community in mouse feces following fecal transplantation from human subjects. **Figure S2.** Pathological analysis of colorectal segments of *Il10*^−/−^ mice following fecal transplantation. **Figure S3.** Cytokine expression of colon in individual groups of mice. **Figure S4.** Quantitation of *E. faecium* in mice by culture after fecal transplantation. **Figure S5.** Quantitation of *E. faecium* and *E. faecalis* in mice by PCR after fecal transplantation. **Figure S6.** Quantitation of *E. faecium* in mice by culture after *E. faecium* inoculation. **Figure S7.** Effect of inoculation with UC-derived or probiotic *E. faecium* strain on colitis. **Figure S8.** Intracellular cytokine staining of colon lamina propria cell after inoculation of *E. faecium* strain IB51a.

**Additional file 3: Figure S9.** Hierarchical clustering analysis of the 10 *E. faecium* strains based on 1683 identified genes.

**Additional file 4: Figure S10.** Bile salt hydrolase (BSH) activity and reactive oxygen species (ROS)-generating ability in *E. faecium* strains. **Figure S11.** Presence of *E. faecium* in feces of UC patients on treatment is not associated with disease activity.

**Additional file 5:.** Review history.

## Data Availability

Sequencing data and metadata were archived in the NCBI Sequence Read Archive under BioProject numbers PRJNA511372 [58] and PRJNA511382 [59].
