# Supplementary material for: Gut-derived Enterococcus faecium from ulcerative colitis patients promotes colitis in a genetically susceptible mouse host
Source: Genome Biol. 2019 Nov 25;20:252. doi: 10.1186/s13059-019-1879-9 (PMC6876129; doi:10.1186/s13059-019-1879-9)
Supplement: Supplementary file 1 — Additional file 1: Table S1. Baseline demographic characteristics of the subjects. Table S2. Disease characteristics of the UC patients. Table S3. Disease characteristics of the CD patients. Table S4. Comparison of KEGG pathway abundance in fecal microbiota between HD and UC subjects by linear. Table S5. Comparison of KEGG pathway abundance in fecal microbiota between HD and CD subjects by linear discriminative analysis. Table S6. Correlation between abundance of fecal Enterococcus and pathology/cytokine production. Table S7. Scoring system for histological evaluation of mouse colon tissues. [file 13059_2019_1879_MOESM1_ESM.docx]

**Additional file 1: Table S1.** Baseline demographic characteristics of the subjects.

|  | HD  (n=13) | CD  (n=8) | UC  (n=16) | *P* value  (HD vs.CD, HD vs. UC, CD vs. UC) |
| --- | --- | --- | --- | --- |
| Gender (M/F) * | 7/6 | 7/1 | 12/4 | NS |
| Age (years) ^†^ | 51.9 ± 12.5 | 33.4 ± 10.6 | 49.4 ± 14.3 | 0.0014, NS, 0.003 |
| BMI (kg/m^2^) ^†^ | 21.6 ± 1.64 | 20.5 ± 3.15 | 20.8 ± 2.90 | NS |
| WBC (/μl) ^†^ | 5069 ± 1574 | 6469 ± 1949 | 6151 ± 3373 | NS |
| Hb (g/dl) ^†^ | NA | 14.2 ± 2.19 | 12.9 ± 2.65 | NS |
| CRP (mg/dl) ^†^ | 0.03 ± 0.03 | 1.22 ± 1.75 | 1.54 ± 3.71 | 0.046, NS, NS |
| Alb (g/dl) ^†^ | NA | 3.90 ± 0.79 | 3.93 ± 0.80 | NS |

^†^, Values are means ± SD. *, Data were analyzed by Chi-squared test. Other data were analyzed by Mann-Whitney U test. Differences were considered significant at *P* < 0.05. BMI, body mass index; WBC, white blood cells; Hb, hemoglobin; CRP, C-reactive protein; Alb, albumin; NS, not significant; NA, not available.

**Additional file 1: Table S2.** Disease characteristics of the UC patients.

|  | n=16 (%) |
| --- | --- |
| Extent of disease |  |
| E1 proctitis | 0 (0%) |
| E2 left-sided colitis | 11 (68.8%) |
| E3 pancolitis | 5 (31.3%) |
| Treatment |  |
| Mesalazine | 14 (87.5％) |
| Corticosteroid | 1 (6.3％) |
| Tacrolimus | 2 (12.5％) |
| TNF antagonist | 4 (25％) |
| Azathioprine / Mercatopurine | 5 / 1 (31.3% / 6.3％) |
| Disease activity |  |
| Total Mayo score (mean and range) | 3.25 (0-11) |

**Additional file 1: Table S3.** Disease characteristics of the CD patients.

|  | n=8 (%) |
| --- | --- |
| Extent of disease (Montreal classification) |  |
| L1 ileal | 2 (25%) |
| L2 colonic | 1 (12.5%) |
| L3 ileocolonic | 4 (50%) |
| L4 isolated upper disease | 1 (12.5%) |
| Behavior |  |
| B1 non structuring, non penetrating | 3 (37.5%) |
| B2 structuring | 4 (50%) |
| B3 penetrating | 1 (12.5%) |
| Treatment |  |
| Mesalazine | 7 (87.5%) |
| Corticosteroid | 1 (12.5%) |
| TNF antagonist | 4 (50%) |
| Elemental diet | 2 (25%) |
| Azathioprine / Mercaptopurine | 3 / 0 (37.5% / 0%) |
| CDAI (mean and range) | 71.1 (0-265) |

CDAI, Crohn’s disease activity index.

**Additional file 1: Table S4.** Comparison of KEGG pathway abundance in fecal microbiota between HD and UC subjects by linear discriminative analysis.

| ko number | class | LDA score (log) | *P* value | Pathway name |
| --- | --- | --- | --- | --- |
| ko00564 | HD | 3.02 | 0.00005 | Glycerophospholipid metabolism |
| ko03410 | UC | 2.81 | 0.00045 | Base excision repair |
| ko00790 | UC | 3.21 | 0.00063 | Folate biosynthesis |
| ko04626 | HD | 2.75 | 0.00073 | Plant-pathogen interaction |
| ko00440 | UC | 2.74 | 0.00101 | Phosphonate and phosphinate metabolism |
| ko00908 | UC | 2.78 | 0.00101 | Zeatin biosynthesis |
| ko00311 | UC | 2.72 | 0.00169 | Penicillin and cephalosporin biosynthesis |
| ko04974 | UC | 2.53 | 0.00247 | Protein digestion and absorption |
| ko00630 | HD | 2.75 | 0.00248 | Glyoxylate and dicarboxylate metabolism |
| ko01051 | HD | 3.83 | 0.00248 | Biosynthesis of ansamycins |
| ko03010 | HD | 3.45 | 0.00380 | Ribosome |
| ko00520 | UC | 2.80 | 0.00501 | Amino sugar and nucleotide sugar metabolism |
| ko00730 | HD | 3.11 | 0.00501 | Thiamine metabolism |
| ko03018 | HD | 2.73 | 0.00967 | RNA degradation |
| ko00052 | UC | 2.78 | 0.01098 | Galactose metabolism |
| ko03030 | UC | 2.81 | 0.01098 | DNA replication |
| ko00910 | UC | 2.56 | 0.01243 | Nitrogen metabolism |
| ko03020 | HD | 2.88 | 0.01243 | RNA polymerase |
| ko00071 | HD | 2.95 | 0.01392 | Fatty acid degradation |
| ko00040 | UC | 2.81 | 0.01406 | Pentose and glucuronate interconversions |
| ko03430 | UC | 2.83 | 0.01406 | Mismatch repair |
| ko00130 | UC | 2.60 | 0.01587 | Ubiquinone and other terpenoid-quinone biosynthesis |
| ko00511 | UC | 3.31 | 0.01587 | Other glycan degradation |
| ko02030 | HD | 3.42 | 0.01762 | Bacterial chemotaxis |
| ko00471 | UC | 3.16 | 0.01788 | D-Glutamine and D-glutamate metabolism |
| ko02010 | UC | 2.46 | 0.01788 | ABC transporters |
| ko03060 | UC | 2.84 | 0.01788 | Protein export |
| ko00450 | HD | 3.25 | 0.01884 | Selenocompound metabolism |
| ko00710 | HD | 2.96 | 0.02076 | Carbon fixation in photosynthetic organisms |
| ko02040 | HD | 3.30 | 0.02112 | Flagellar assembly |
| ko00720 | UC | 3.02 | 0.02259 | Carbon fixation pathways in prokaryotes |
| ko00785 | UC | 3.18 | 0.02259 | Lipoic acid metabolism |
| ko00510 | UC | 2.63 | 0.02389 | N-Glycan biosynthesis |
| ko00121 | HD | 3.51 | 0.02532 | Secondary bile acid biosynthesis |
| ko00480 | UC | 2.57 | 0.03929 | Glutathione metabolism |
| ko00250 | HD | 2.73 | 0.04598 | Alanine, aspartate and glutamate metabolism |
| ko00100 | HD | 2.68 | 0.04653 | Steroid biosynthesis |
| ko03022 | HD | 2.90 | 0.04653 | Basal transcription factors |

Linear discriminative analysis (LDA) was performed using linear discriminative analysis with effect size (LEfSe) to identify significant differences in relative abundance of KEGG pathways in the feces of HD subjects compared with UC subjects. LDA scores with *P*-values < 0.05 are shown in the table.

**Additional file 1: Table S5.** Comparison of KEGG pathway abundance in fecal microbiota between HD and CD subjects by linear discriminative analysis.

| ko number | class | LDA score (log10) | *P* value | Pathway name |
| --- | --- | --- | --- | --- |
| ko00564 | HD | 3.04 | 0.00051 | Glycerophospholipid metabolism |
| ko00970 | HD | 3.34 | 0.00051 | Aminoacyl-tRNA biosynthesis |
| ko00750 | HD | 3.01 | 0.00112 | Vitamin B6 metabolism |
| ko04626 | HD | 2.77 | 0.00144 | Plant-pathogen interaction |
| ko00510 | CD | 2.86 | 0.00175 | N-Glycan biosynthesis |
| ko00052 | CD | 2.89 | 0.00235 | Galactose metabolism |
| ko00130 | CD | 2.87 | 0.00235 | Ubiquinone and other terpenoid-quinone biosynthesis |
| ko00053 | CD | 3.05 | 0.00249 | Ascorbate and aldarate metabolism |
| ko00790 | CD | 3.20 | 0.00299 | Folate biosynthesis |
| ko03018 | HD | 2.79 | 0.00299 | RNA degradation |
| ko00785 | CD | 3.19 | 0.00377 | Lipoic acid metabolism |
| ko03010 | HD | 3.58 | 0.00377 | Ribosome |
| ko00290 | HD | 3.35 | 0.00474 | Valine, leucine and isoleucine biosynthesis |
| ko00730 | HD | 3.30 | 0.00737 | Thiamine metabolism |
| ko00330 | HD | 2.63 | 0.01125 | Arginine and proline metabolism |
| ko00311 | CD | 2.64 | 0.01424 | Penicillin and cephalosporin biosynthesis |
| ko00040 | CD | 2.94 | 0.01685 | Pentose and glucuronate interconversions |
| ko00440 | CD | 2.69 | 0.01685 | Phosphonate and phosphinate metabolism |
| ko00480 | CD | 2.75 | 0.01685 | Glutathione metabolism |
| ko00140 | CD | 2.78 | 0.01766 | Steroid hormone biosynthesis |
| ko00310 | CD | 2.74 | 0.01894 | Lysine degradation |
| ko00450 | HD | 2.95 | 0.02032 | Selenocompound metabolism |
| ko00360 | CD | 2.66 | 0.02044 | Phenylalanine metabolism |
| ko00230 | HD | 2.67 | 0.02048 | Purine metabolism |
| ko01051 | HD | 3.76 | 0.02048 | Biosynthesis of ansamycins |
| ko03020 | HD | 2.94 | 0.02477 | RNA polymerase |
| ko00540 | CD | 3.11 | 0.02960 | Lipopolysaccharide biosynthesis |
| ko00121 | HD | 3.59 | 0.02981 | Secondary bile acid biosynthesis |
| ko00620 | HD | 2.91 | 0.02981 | Pyruvate metabolism |
| ko00400 | HD | 2.78 | 0.03571 | Phenylalanine, tyrosine and tryptophan biosynthesis |
| ko00770 | HD | 3.00 | 0.03571 | Pantothenate and CoA biosynthesis |
| ko00562 | CD | 2.73 | 0.04197 | Inositol phosphate metabolism |
| ko00511 | CD | 3.31 | 0.04258 | Other glycan degradation |
| ko00071 | HD | 2.83 | 0.04635 | Fatty acid degradation |
| ko00906 | HD | 3.00 | 0.04670 | Carotenoid biosynthesis |

LDA was performed using LEfSe to identify significant differences in the relative abundance of KEGG pathways in the feces of HD subjects compared with CD subjects. LDA scores with *P*-values < 0.05 are shown in the table.

**Additional file 1: Table S6.** Correlation between abundance of fecal *Enterococcus* and pathology/cytokine production.

|  |  | Pathology score | tnf | il1b | il6 | il12b | il17a | il23a |
| --- | --- | --- | --- | --- | --- | --- | --- | --- |
| %Enterococcus genus  (16SrRNA-based sequencing) | r P-value | 0.4832 0.0021 | 0.3917 0.015 | 0.4184 0.0089 | 0.4821 0.0022 | 0.2151 0.1947 | 0.5929 <0.0001 | -0.02988 0.8587 |
| Copy number of *E. faecium*  (qPCR) | r P-value | 0.2456 0.1373 | 0.09847 0.5564 | 0.2838 0.0842 | 0.2852 0.0827 | 0.3026 0.0648 | 0.3416 0.0358 | 0.1634 0.3269 |
| Copy number of *E. faecalis*  (qPCR) | r P-value | 0.3025 0.0649 | 0.1925 0.2469 | 0.2723 0.0981 | 0.3194 0.0506 | 0.2363 0.1532 | 0.3425 0.0353 | 0.1845 0.2673 |
| Copy number of (*E. faecium* + *E. faecalis*)  (qPCR) | r P-value | 0.4443 0.0052 | 0.2154 0.1941 | 0.3895 0.0156 | 0.3527 0.0299 | 0.3626 0.0253 | 0.4435 0.0053 | 0.191 0.2507 |

Data were analyzed by Spearman’s rank correlation. qPCR, quantitative PCR; r, correlation coefficient.

**Additional file 1: Table S7.** Scoring system for histological evaluation of mouse colon tissues.

| Score | Mucosa (M) | Inflammation (I) | Extent (E) * |
| --- | --- | --- | --- |
| 0 | No significant lesions | None | No significant changes |
| 1 | Mild epithelial hyperplasia | Mild inflammation limited to mucosa | <1% of segment affected |
| 2 | Moderate epithelial hyperplasia | Moderate inflammation in mucosa and submucosa | 1-30% of segment affected |
| 3 | Severe epithelial hyperplasia with crypt branching or herniation | Severe inflammation with obliteration of normal architecture, erosions, or crypts abscessed | 1-60% of segment affected |
| 4 |  | Level 3 changes + ulceration | >60% of segment affected |

Segment score = M + I + E1 + E2. Pathology score = sum of the segment scores of proximal colon, distal colon, and rectum.

*E1 = % of segment affected in any manner; E2 = % of segment with level 3 or 4 changes in M or I score.
