# Supplementary material for: Gut-derived Enterococcus faecium from ulcerative colitis patients promotes colitis in a genetically susceptible mouse host
Source: Genome Biol. 2019 Nov 25;20:252. doi: 10.1186/s13059-019-1879-9 (PMC6876129; doi:10.1186/s13059-019-1879-9)
Supplement: Supplementary file 2 — Additional file 2: Figure S1. β-Diversity analysis of the bacterial community in mouse feces following fecal transplantation from human subjects. Figure S2. Pathological analysis of colorectal segments of Il10−/− mice following fecal transplantation. Figure S3. Cytokine expression of colon in individual groups of mice. Figure S4. Quantitation of E. faecium in mice by culture after fecal transplantation. Figure S5. Quantitation of E. faecium and E. faecalis in mice by PCR after fecal transplantation. Figure S6. Quantitation of E. faecium in mice by culture after E. faecium inoculation. Figure S7. Effect of inoculation with UC-derived or probiotic E. faecium strain on colitis. Figure S8. Intracellular cytokine staining of colon lamina propria cell after inoculation of E. faecium strain IB51a. [file 13059_2019_1879_MOESM2_ESM.pdf]

Additional file 2: Fig. S1

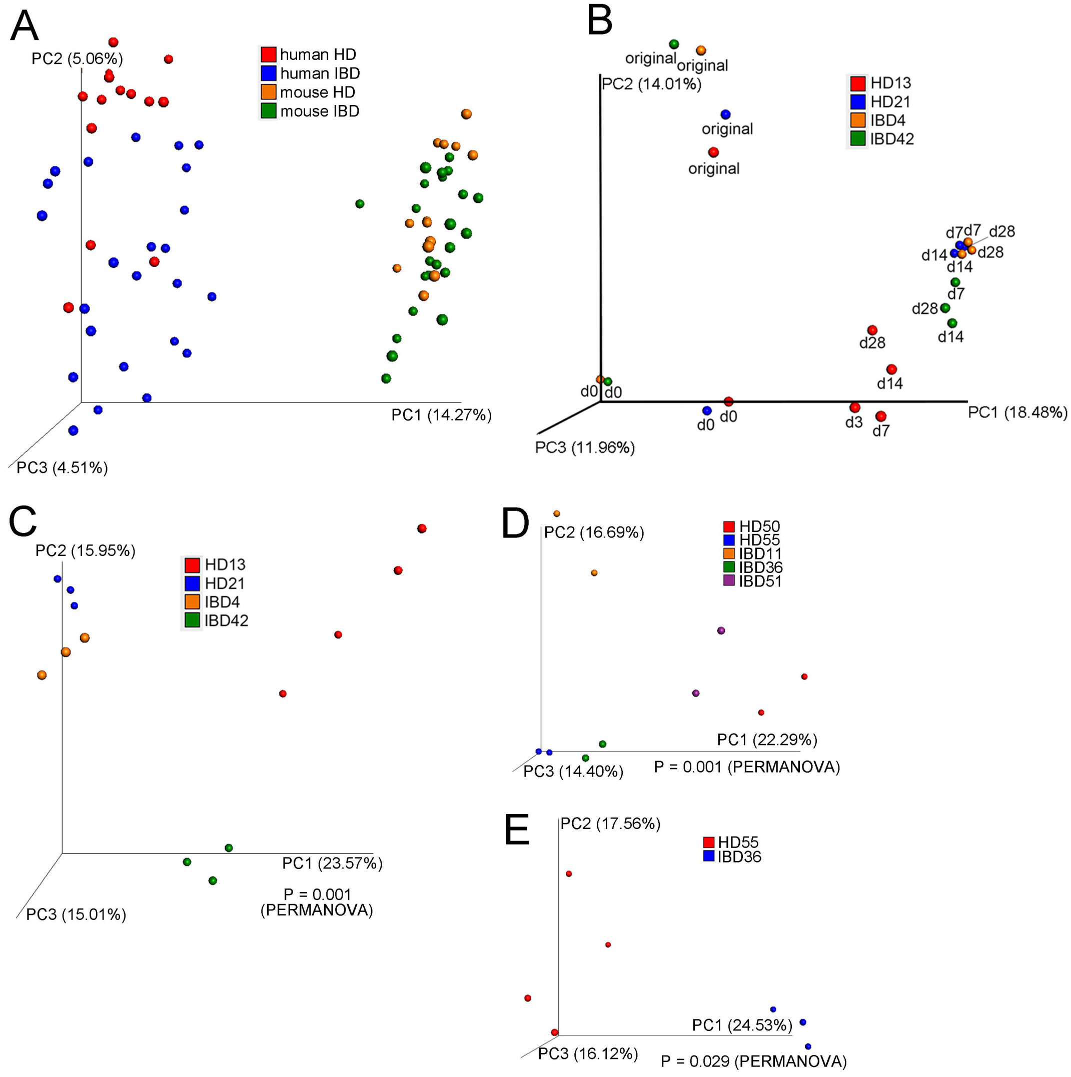

β-Diversity analysis of the bacterial community in mouse feces following fecal transplantation from human subjects. Unweighted UniFrac distances were calculated from the taxonomic data derived from 16S rRNA-based metagenomes of human or mouse feces and visualized as PCoA plots. (a) 16S rRNA-based sequencing data for 37 human subjects, including 13 HD and 16 UC and 8 CD patients, as well as sequencing data for 37 corresponding *Il10*$^{-/-}$ mice that received transplantation from each subject, were used to generate PCoA plots. (b and c) b shows PCoA plots derived from metagenomes of *Il10*$^{-/-}$ mice that received transplantation of feces from individual subjects HD13, HD21, IBD4, or IBD42 at various time points following fecal transplantation as well as those from the original human subjects. In c, only the metagenomes after transplantation of feces from donors HD13, HD21, IBD4, or IBD42 (day 3, 7, 14 or 28) were analyzed and shown. d, day. (d) The indicated five fecal samples were transplanted into two cages of mice (male or female) per fecal donor. To see compositional differences between the two cages, PCoA plots based on unweighted UniFrac distances were visualized. (e) Feces from donors HD55 or IBD36 was independently transplanted into mice 4 or 3 times, respectively. To see compositional differences caused by repeats of the transplantation, PCoA plots based on unweighted UniFrac distances were visualized. Significant differences between subject-based groups in c, d and e were determined by PERMANOVA and P-values are shown at the bottom of the plots.

Additional file 2: Fig. S2

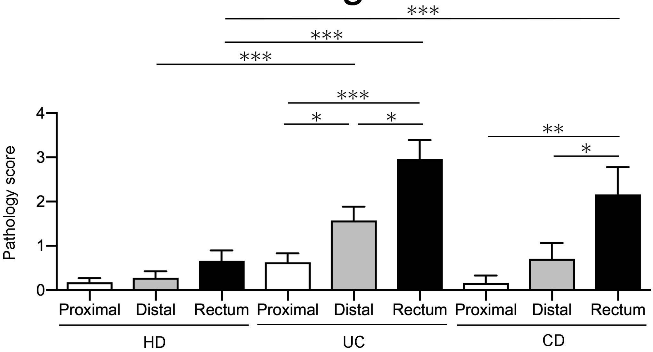

Pathological analysis of colorectal segments of *Il10*$^{-/-}$ mice following fecal transplantation. Fecal suspensions from HD, UC, or CD subjects were transplanted into flora-depleted *Il10*$^{-/-}$ mice as described in Figure 2. Pathology scores for each segment, including the proximal colon, distal colon, and rectum, were calculated separately. Statistical differences between two values were analyzed using a Mann-Whitney U test. *, $P < 0.05$; **, $P < 0.01$; ***, $P < 0.001$.

Additional file 2: Fig. S3

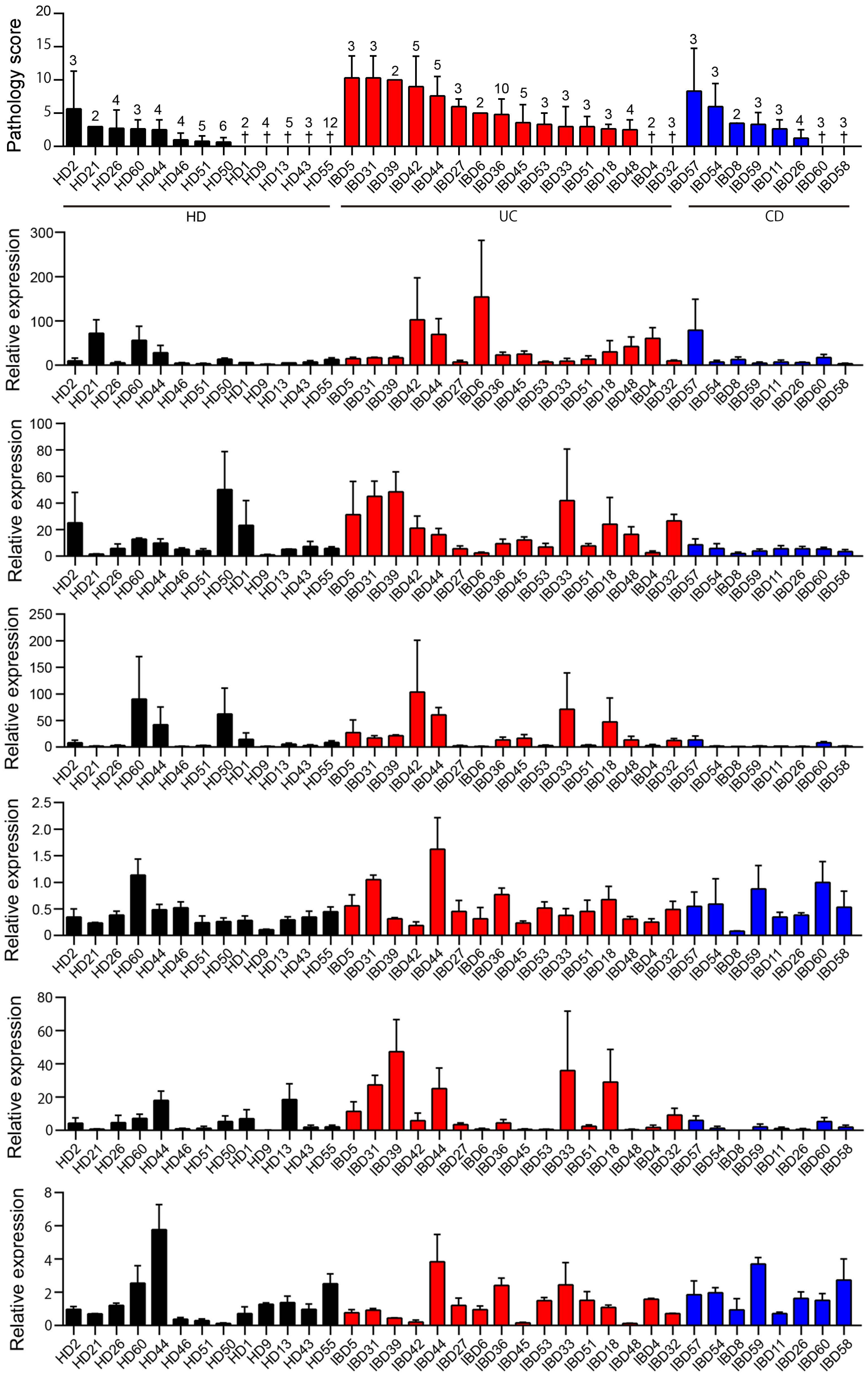

Cytokine expression of colon in individual groups of mice. Mean cytokine expression in mouse colon corresponding to individual fecal donors. The identification codes of individual subjects are shown on the X-axis, and correspond to those in Figure 2. Mean ± SE. The numbers above the error bars indicate the number of mice in each category.

Additional file 2: Fig. S4

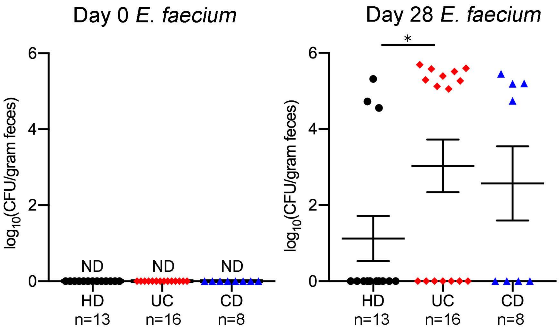

Quantitation of *E. faecium* in mice by culture after fecal transplantation. Colony-forming units (CFU) of *E. faecium* in feces of mice before or 28 days after fecal transplantation. Feces of representative mice corresponding to the 37 subjects was examined. Mean ± SE. *, $P < 0.05$.

Additional file 2: Fig. S5

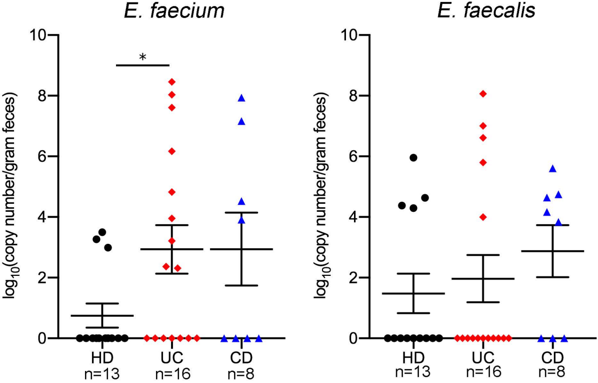

Quantitation of *E. faecium* and *E. faecalis* in mice by PCR after fecal transplantation. Copy number of *E. faecium* (left) or *E. faecalis* (right) in feces of mice after fecal transplantation. Feces of representative mice corresponding to the 37 subjects was examined. Mean ± SE. *, $P < 0.05$.

Additional file 2: Fig. S6

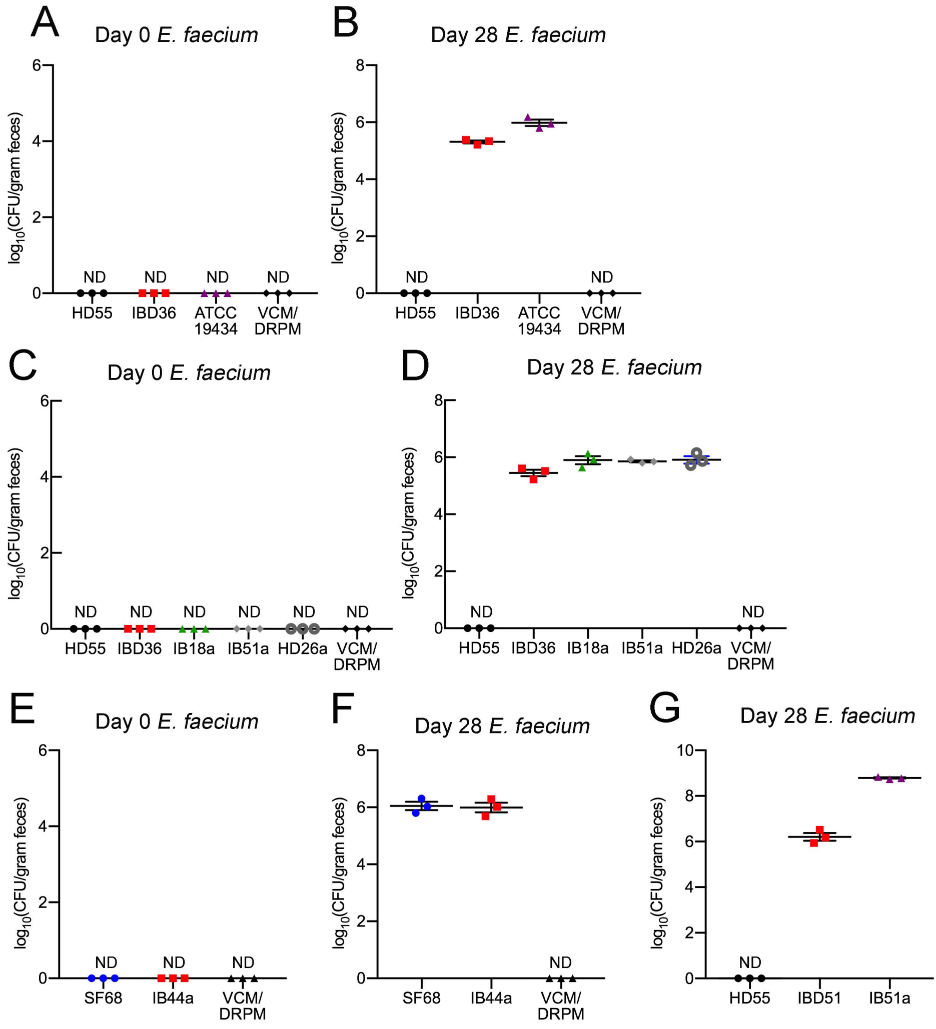

Quantitation of *E. faecium* in mice by culture after *E. faecium* inoculation. CFU of *E. faecium* in feces of mice before or 28 days after fecal transplantation or inoculation of the indicated *E. faecium* strain. Feces of three representative mice from each group was examined. Mean ± SE. ND, not detected.

Additional file 2: Fig. S7

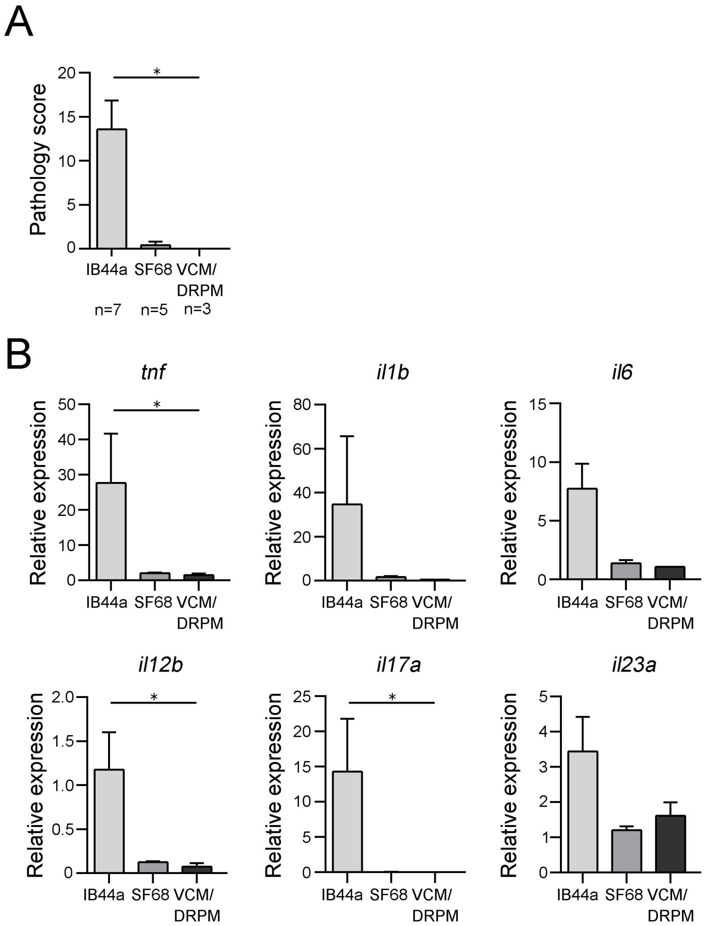

Effect of inoculation with UC-derived or probiotic *E. faecium* strain on colitis. UC-derived *E. faecium* strain IB44a or probiotic *E. faecium* strain SF68 was inoculated into flora-depleted *Il10*$^{-/-}$ mice. The control group was treated with antibiotics (VCM/DRPM) in the absence of transplantation. (a) Mean pathology scores of mice from each treatment group. (b) mRNA expression levels of inflammatory cytokines in the colon analyzed by real-time PCR. Statistical differences between a value and the microbiota-depleted control were analyzed using the Kruskal-Wallis test followed by Dunn’s test. Mean ± SE. *, $P < 0.05$

Additional file 2: Fig. S8

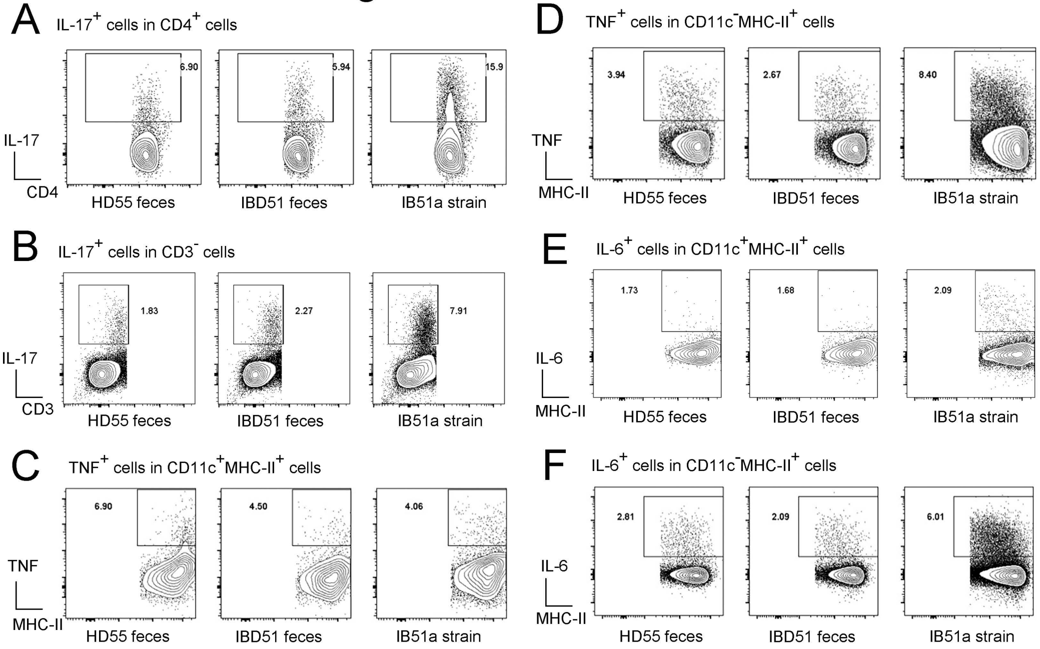

Intracellular cytokine staining of colon lamina propria cells after inoculation of *E. faecium* strain IB51a. This figure is related to Figure 6f. Germ-free *Il10*$^{-/-}$ mice received fecal transplantation from donor HD55 or IBD51, or were inoculated with *E. faecium* strain IB51a. After 21 days, single cells were obtained from colonic lamina propria and stained with antibodies to the indicated cytokines. Percentages of cytokine-positive cells are shown in the plots.
