# Supplementary figures and images for: Gut-derived Enterococcus faecium from ulcerative colitis patients promotes colitis in a genetically susceptible mouse host
Source: Genome Biol. 2019 Nov 25;20:252. doi: 10.1186/s13059-019-1879-9 (PMC6876129; doi:10.1186/s13059-019-1879-9)

### Additional Figure 9

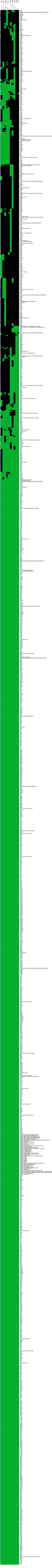

Supplement: Supplementary file 3 — Additional file 3: Figure S9. Hierarchical clustering analysis of the 10 E. faecium strains based on 1683 identified genes. [file 13059_2019_1879_MOESM3_ESM.pdf]
