# Supplementary material for: Gut-derived Enterococcus faecium from ulcerative colitis patients promotes colitis in a genetically susceptible mouse host
Source: Genome Biol. 2019 Nov 25;20:252. doi: 10.1186/s13059-019-1879-9 (PMC6876129; doi:10.1186/s13059-019-1879-9)
Supplement: Supplementary file 4 — Additional file 4: Figure S10. Bile salt hydrolase (BSH) activity and reactive oxygen species (ROS)-generating ability in E. faecium strains. Figure S11. Presence of E. faecium in feces of UC patients on treatment is not associated with disease activity. [file 13059_2019_1879_MOESM4_ESM.pdf]

Additional file 4: Fig. S10

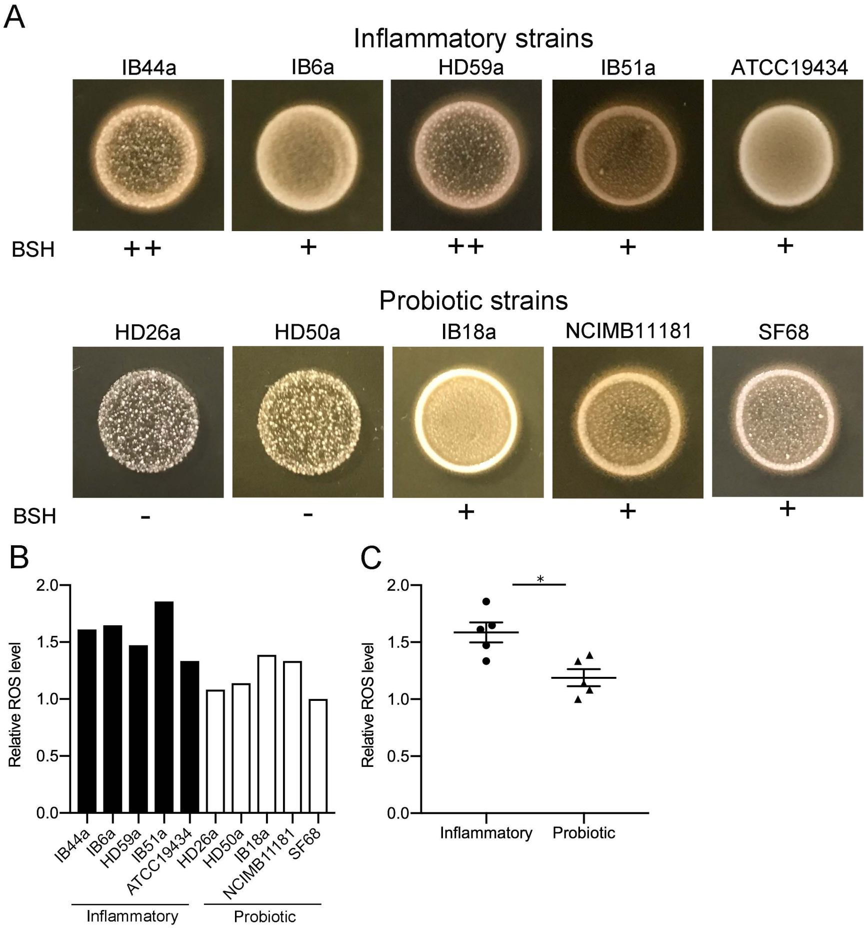

Bile salt hydrolase (BSH) activity and reactive oxygen species (ROS)-generating ability in *E. faecium* strains. (a) BSH activity of 10 *E. faecium* strains was evaluated in culture with glyco-conjugated deoxycholic acid (GDCA)-containing agar. If white zonal change of DCA appeared around colonies, BSH activity was considered positive. ++, the thickness of zone ≥ 1.0 mm; +, 0 < the thickness < 1.0 mm; -, no zonal change.

(b and c) Supernatant of culture with *E. faecium* strains was evaluated for presence of ROS. Relative ROS level in individual strains (b) and average ROS level in the inflammatory or the probiotic cluster (c) are shown. Mean ± SE in (c). *, $P < 0.05$.

Additional file 4: Fig. S11

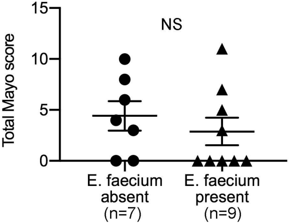

Presence of *E. faecium* in feces of UC patients on treatment is not associated with disease activity. Total Mayo score of 16 UC patients is shown according to the presence of *E. faecium* in feces determined by quantitative PCR. NS, not significantly different.
