# Supplementary material for: Gut-derived Enterococcus faecium from ulcerative colitis patients promotes colitis in a genetically susceptible mouse host
Source: Genome Biol. 2019 Nov 25;20:252. doi: 10.1186/s13059-019-1879-9 (PMC6876129; doi:10.1186/s13059-019-1879-9)
Supplement: Supplementary file 5 — Additional file 5:. Review history. [file 13059_2019_1879_MOESM5_ESM.docx]

Review History

**First round of review**

**Reviewer 1**

**Are you able to assess all statistics in the manuscript, including the appropriateness of statistical tests used?** No, I do not feel adequately qualified to assess the statistics.

**Comments to author:**

The manuscript "Gut-derived Enterococcus faecium from Ulcerative Colitis Patients Promotes Colitis in a Genetically-susceptible Host" by Jun Seishima and colleagues describes the effect of IBD associated microbiota in an antibiotic treated IL10-/- mouse model. Further they isolate E. faecium strains from IBD patients and healthy controls and show IBD-related strains may be pro-inflammatory. Overall I find these findings are very interesting and authors provide quite comprehensive data. My concerns are outlined below

1.     The authors use an antibiotic treated IL10-/- model which makes it difficult to assess the impact of the microbiome given that it results in a hybrid microbiome. As seen in additional figure 1, the IBD and healthy patient microbiome is indistinguishable following colonization of mice, hence it is difficult to draw conclusions. The authors mention all stool samples were used but only show two donors each in healthy and IBD group in additional figure 1. The authors need to provide more information about husbandry. Were mice colonized by individual stool samples housed separately? Did they see a cage effect? The authors should show the microbiome data for all the colonized mice and the human donors which will help address these questions.  In addition there are also questions about reproducibility when using antibiotics as the treatment effects are not necessarily consistent. Did the authors repeat the colonization experiments to assess reproducibility?
2.     The authors show data on individual strains of E. faecium isolated from patients along with colonization with the entire microbiome from UC patient but it is unclear why they chose a different UC microbiome than the ones from which the E. faecium strains were isolated.   It would be more appropriate to use the same stool sample to see if the effect of the entire microbiome can in fact be mimicked by just using a single strain.
3.     The Crohns disease associated microbiome also causes inflammation but it is unclear why the authors don't look at E. faecium isolated from CD patients?  
4.     It will be helpful to know the genes which are different among the E. faecium strains based on genome alignment as it could provide clues for potential mediators of the pro-inflammatory effect.
5.     The paper needs to be edited for language and grammar.

**Reviewer 2**

**Are you able to assess all statistics in the manuscript, including the appropriateness of statistical tests used?** Yes, and I have assessed the statistics in my report.

**Comments to author:**

A deeper understanding of the potential role for microbiota in the pathogenesis of IBD is needed.  In this study, the authors use fecal material from 37 patients (13 HD, 16 UC, 8 CD) to investigate the potential contribution.  Their results show some differences in the microbiome of HD vs. UC and HD vs. CD characterized by E. faecium and E. coli respectively.  The authors subsequently transfer these microbiota into antibiotic pretreated IL10-deficient mice and disease activity was assessed.  In aggregate, both UC and CD microbiota resulted in more severe weight loss and pathologic inflammation (Fig. 2) and this differentially correlated with cytokine production (Fig. 3).  Although engraftment was poorly representative of the donor microbiota (Additional Fig. 1), Enterococcus abundance differentiates HD vs. UC and the high enterococcus samples showed higher cytokine production.  The authors subsequently show that E. faecium was sufficient to trigger colitis and inflammatory cytokines in IL10-deficient mice and that differences exist both phenotypically and genomically in isolates of E. faecium.  This work helps to contribute to the understanding for the potential role of IBD microbiota in the ettiology of colitis, but additional analysis are need to substantiate the potential contribution of E. faecium strains underlying this physiology.

Specific points:
1.  The authors use both inactive and active UC samples but primarily inactive CD samples.  Are differences in the microbial profiles of these samples correlated with disease activity as previously reported (Gevers, et al Cell Host and Microbe 2014)?  Previous reports suggested that donor activity did not impact colitis in a transfer T cell colitis model (Britton et al Immunity 2019).  Does donor disease activity impact IL-10 deficient colitis phenotype (Fig. 2) and cytokine production (Fig. 3)?  What was the rationale for patient sample selection in regards to the fecal transplant mouse models?

Furthermore, it would be helpful to provide analysis of cytokine production on a per donor basis in Figure 3 to assess if certain donors are driving particular immune phenotypes.  In addition, cellular phenotyping by flow cytometry would increase the clarity and impact of these findings.

2.  
A. For the patients' fecal samples subjected to metagenomics whole genome sequencing, what was the sequencing depth for each sample before and after quality control?

B. For the mouse fecal samples subjected to 16S target sequencing, what was the sequencing depth for each sample? Did the authors use any rarefication procedure before beta diversity calculation and taxonomic profiling?

3.  Engraftment of donor microbiota in this model does not appear to be robust (Additional Fig. 1).  

A. Although in the supplemental material figure 1 post FMT did not group closer to the pre FMT fecal samples, they did not group closer to the donor fecal samples either, suggesting that both human and mouse are impacting the post FMT gut microbial community. Furthermore, regardless of the donor health status, the majority of the mouse post FMT samples clustered closer. Therefore, the "community microbiota uniquely dependent on the human subjects' flora" statement is not appropriate. Statistical analysis was not provided to support this statement.

B.  Another concern here to be discussed is the potential to miss key strains that do not engraft.  Furthermore, although Enteroccocus in enriched in UC transplant compared to HD (Fig. 4A), it is not clear if this is donor-derived E. faecium or the strain assignment (as per Fig. 6E) of the engrafted E. faecium strains.

4.  It is interesting that Enteroccocus abundance correlates with cytokine production in the IL10-deficient mice (Fig. 4B).

A.  The median percentage abundance of 0.003% seems very low.  Is there a linear correlation of Enteroccocus with cytokine production to help substantiate the potential correlation?

B.  Is it clear that Enterococcus abundance maps to E. faecium?  Does this association hold at the taxonomic level assignment (as done in Fig. 1A)?

C.  What samples does the N=20 in Fig. 4B refer to?  Is this individual mice or individual donors?  Is this analysis performed with UC and/or CD donors?  What is the effect of enterococcus abundance on cytokine production in HD colonized mice?

5.  The authors show a differential effect of IBD patient-derived and ATCC E. faecium compared to HD-derived E. faecium in Figure 5.

A.  Can the authors confirm colonization (equivalent colonization) of the HD and IBD isolates to assess that this is not reflective of colonization?  This should be provided for all isolate colonization experiments.

B.  Assuming equivalent colonization, can FISH be used to assess spatial location as potential mechanism for these strain differences.

C.  The data indeed suggest that E. faecium plays a role in disease, however the microbiota depleted IL10-/- model does not provide enough evidence to use the "causal" statement. Mouse microbiota can still contribute to the disease phenotype observed in the IBD recipient mice, especially due to the data shown in supplemental figure 1, in which pre FMT microbiota of mice receiving feces from healthy donors seems to be distinct from that of IBD recipient's mice.  These points should be included in the Discussion.

6.  The dichotomy between weight loss / pathology and cytokine production is a bit unclear in both Figures 5 and 6.  For example, why does IBD36 derived strain and ATCC strain induce pathology and weight loss but differences in cytokine?  Is this related to IL10-deficiency mechanistically?  An additional model of colitis to evaluate these strains would help the authors assess this.

7.  The hypothesis that strain variation account for differences in intriguing, but not strongly supported by the data (as stated on page 10, line 5-6).  

A.  The resolution of figure 6 is very low making it difficult to read strain names. It is also difficult to decipher strain nomenclature.

B.  While Fig. 6E supports the possible existence of genomic strains, the correlation with colitis pathology and/or cytokine production is confusing.  While pathogenic ATCC isolates segregates with 51a, disease inducing isolate 18a segregates with probiotic and HD strains.  Moreover, HD59a (unclear due to low resolution of the figure provided) segregates with the ATCC isolate.  These data do not clearly support a meaningful association with function.  Can the authors clarify this?  Mechanistic data showing a strain effect (suggested in the Discussion) would be ideal.  It would be helpful at least to provide colitis and cytokine data for each isolate profiled in Fig. 6E as well as confirmation of colonization.

C.  Error bars in Figure 6C seem larger than would be expected with 2 and 3 star p-value significance.  Can the authors clarify this finding? Did the authors apply any multiple comparison correction in the analyses described in figure 6?

8.  The authors suggest that E. faecium presence correlates with disease extent and combination therapy.

A.  For the N=5 with E. faecium, does E. faecium abundance linearly correlate with disease activity score?

B.  For the N=5 with E. faecium, does genomic analysis suggest a particular strain type?

C.  It is interesting that the authors focused on E. faecium finding if only present in 5/16 UC patients.  What is driving colitis in 11/16 samples if not E. faecium (in Fig. 2A)?  Also, what is the source of the Enterococcus seen in Figure 4, particularly for the samples from donors without Enterococcus?

**Authors Response**

**Point-by-point responses to the reviewers’ comments:**

*Response to Reviewer 1: Thank you for your review of our paper. We have answered each of your points below. Line numbers refer to the new version of the paper.*

Comment 1: The authors use an antibiotic treated IL10-/- model which makes it difficult to assess the impact of the microbiome given that it results in a hybrid microbiome. As seen in additional figure 1, the IBD and healthy patient microbiome is indistinguishable following colonization of mice, hence it is difficult to draw conclusions. The authors mention all stool samples were used but only show two donors each in healthy and IBD group in additional figure 1. The authors need to provide more information about husbandry. Were mice colonized by individual stool samples housed separately? Did they see a cage effect? The authors should show the microbiome data for all the colonized mice and the human donors which will help address these questions. In addition there are also questions about reproducibility when using antibiotics as the treatment effects are not necessarily consistent. Did the authors repeat the colonization experiments to assess reproducibility?

*Response: We have modified the Figures and text to answer the Comment 1 as follows:*

*(1)We agree that murine microbiota derived from HD feces and IBD feces generated mixed clusters in PCoA analysis in new Additional Figure 1A. However, mouse microbiota derived from a single donor closely clustered even 28 days posttransplantation and segregated from the microbiota derived from other donors with significant statistical difference by PERMANOVA, as shown in new Additional Figure 1C. Effects of cage difference (Additional Figure 1D) and repeats of transplantation (Additional Figure 1E) on the compositional change of gut microbiota were modest, and the murine microbiota were significantly segregated by difference of the fecal donor (Additional Figure 1D, P = 0.001; Additional Figure 1E, P = 0.029: PERMANOVA).*

*(2)Microbiome data for all the colonized mice and the human donors are shown in new Additional Figure 1A.*

*(3)Mice colonized by individual stool samples were housed separately, as described in METHODS, page 17, line 19.*

*In summary, transplantation of the subjects’ feces created subject-unique microbiota in Il10−/− mice and the composition of microbiota was different from other-donor-derived microbiota with very modest effects of cage difference and repeats of transplantation. Therefore, by analyzing the mouse microbiota after fecal transplantation, we could find associations between colitis and a specific microbe. Although the mouse microbiota did not represent complete restoration of human microbiota, by comparing human and mice microbiota and performing the following culture and single strain experiments, we found that patient-derived E. faecium can promote colitis.*

*We added new PCoA results in Additional Figure 1 and describe them in RESULTS, page 6, line 17.*

Comment 2: The authors show data on individual strains of E. faecium isolated from patients along with colonization with the entire microbiome from UC patient but it is unclear why they chose a different UC microbiome than the ones from which the E. faecium strains were isolated. It would be more appropriate to use the same stool sample to see if the effect of the entire microbiome can in fact be mimicked by just using a single strain.

*Response: To examine the effect of inoculation of a single E. faecium strain on colitis in Figures 5 and 6, we chose HD55 feces and IBD36 feces as negative and positive controls for inflammation, respectively. As pointed out in Comment 2, they were “inflammation controls” and not “microbiota controls”. Thus, in new Figures 6E and F, we compared IBD51 feces-transplanted and E. faecium strain IB51a-inoculated gnotobiotic mice. Both IBD51 feces-transplanted mice and IB51a-colnized mice showed more severe colitis than HD55 feces-transplanted controls. However, the increased cytokine expression was different between the IBD51 feces and IB51a strain groups. Interaction between other bacterial species and E. faecium strain IB51a may result in a different pattern of cytokine expression. As an answer to Comment 2, we can say that inoculation of single strain IB51a mimicked pathological colitis following IBD51 fecal transplantation.*

*These experiments are described in RESULTS, page 10, line 5.*

Comment 3: The Crohns disease associated microbiome also causes inflammation but it is unclear why the authors don't look at E. faecium isolated from CD patients?

*Response: In this study we focused on UC, not CD, based on results of Figure 1B which revealed that E. faecium or E. coli was most discriminative between UC and HD, and CD and HD, respectively. Additional Figure 5 did show that after fecal transplantation from CD subjects, a few mice had E. faecium in their microbiota. We agree that E. faecium might also have a pathological role in CD patients and this needs to be examined in the future. However, we decided that UC is more associated with E. faecium than CD based on our existing data, and examined the effect of E. faecium strains from UC patients on colitis in this study.*

Comment 4: It will be helpful to know the genes which are different among the E. faecium strains based on genome alignment as it could provide clues for potential mediators of the pro-inflammatory effect.

*Response: We identified the presence of KEGG-based pathways in the genome of 10 E. faecium strains (shown in new Figure 7A). Then, significantly discriminative pathways between the “inflammatory cluster” (including strains IB44a, IB6a, HD59a, IB51a and ATCC19434) and the “probiotic cluster” (including strains HD50a, HD26a, IB18a, NCIMB11181 and SF68) were identified by linear discriminative analysis with LEfSe. Four pathways were significantly discriminative, as shown in Figure 7B. Among those pathways, secondary bile acid biosynthesis, which increased in the inflammatory cluster, might be associated with colitis, because particular bile acids control biofilm formation of Enterococcus, polarization of host macrophages, and generation of oxidative stress in the colon.*

*Based on these results, we created new Figure 7B and modified RESULTS, page 10, line 28, and DISCUSSION, page 15, line 6. Old Figure 6E was moved to become new Figure 7A.*

Comment 5: The paper needs to be edited for language and grammar.

*Response: The revised manuscript was edited by a Edanz Ltd. for language and grammar.*

*Response to Reviewer 2: Thank you for your review of our paper. We have answered each of your points below. Line numbers refer to the new version of the paper.*

Comment 1: The authors use both inactive and active UC samples but primarily inactive CD samples. Are differences in the microbial profiles of these samples correlated with disease activity as previously reported (Gevers, et al Cell Host and Microbe 2014)? Previous reports suggested that donor activity did not impact colitis in a transfer T cell colitis model (Britton et al Immunity 2019). Does donor disease activity impact IL-10 deficient colitis phenotype (Fig. 2) and cytokine production (Fig. 3)? What was the rationale for patient sample selection in regards to the fecal transplant mouse models?

*Response: We stated criteria for enrollment of the IBD subjects in the METHODS section, page 16, line 3, as follows:*

*This study aimed to examine fecal microbiota of IBD patients who visited Kanazawa University Hospital in various stages including both active and remission stages. Between February 2014 and March 2015, 60 IBD patients enrolled in the study. Among them 55 subjects received colonoscopy to evaluate disease status. To investigate the composition and metagenomes of the gut microbiota of the different patient groups, fecal samples were collected from patients immediately after enrollment and stored at −80°C. Among the 55 subjects, 16 UC patients and 8 CD patients provided us with good and sufficient feces immediately after enrollment, and these feces were used for the study. Feces that were too watery or that were left at room temperature for >3 h were not used.*

*The CD subjects included one active patient (CDAI >150) and seven inactive patients (CDAI < 150). Twenty-three of the 24 IBD patients had already received treatment, so disease activity was fundamentally controlled by treatment. Therefore, although E. faecium was present at a significantly higher level in UC patients (new Figure 1E), the presence of fecal E. faecium was not associated with Mayo disease activity of patients in this study (new Additional Figure 10). If samples were collected in a treatment-naïve state, disease activity may be associated with microbiota, as shown in a previous report (Gevers et al. Cell Host and Microbe, 2014). In contrast, disease type (pancolitis or left-sided colitis) is usually not changed even after treatment is started. Frequency of pancolitis was higher in E. faecium-colonized UC patients in a non-treatment-naïve state (Figure 8A). A requirement for combination treatments indicates resistance to a single treatment, and is a good marker for clinical disease status of non-treatmentnaïve patients. In our analysis, the use of multiple medications per patient was positively associated with the presence of E. faecium in feces (Figure 8B). Upon fecal transplantation, mice were assessed for the effect of fecal microbiota composition on colitis, independently of disease activity of patients, because (unlike in the humans) the mice did not receive any treatment.*

*We modified the RESULTS text related to new Figure 8A and 8B and Additional Figure 10, as shown on page 11, lines 12 to 20.*

Comment 1-B: Furthermore, it would be helpful to provide analysis of cytokine production on a per donor basis in Figure 3 to assess if certain donors are driving particular immune phenotypes. In addition, cellular phenotyping by flow cytometry would increase the clarity and impact of these findings.

*Response: We determined cytokine production on a per donor basis and now show this in Additional Figure 3 and in RESULTS, page 7, line 19. Cellular phenotyping by flow cytometry was performed with colon lamina propria cells of HD55-feces- or IBD51-feces-transplanted, or IB51a-inoculated gnotobiotic mice, as shown in Additional Figure 8. IL-17 was produced by both Th17 cells and a CD3- negative population. TNF and IL-6 were produced mainly by MHC class-II-positive CD11c-negative cells, as well as by a smaller proportion of MHC class-II-positive CD11c-positive cells.*

*Please see RESULTS, page 10, line 12. We have also added new Additional METHODS text regarding the flow cytometry. The sentence “and the UC- or the CD-microbiota tended to direct to expression of Il6/Il17a or Il23a, respectively, at least in this model” was deleted from RESULTS, page 7, line 22, because it was not necessarily true based on cytokine expression in this gnotobiotic experiment.*

*We also added a new coauthor, Akihiro Seki, because he performed the flow cytometry experiments.*

Comment 2-A: For the patients' fecal samples subjected to metagenomics whole genome sequencing, what was the sequencing depth for each sample before and after quality control?

*Response: The average number of reads per sample before and after quality control was 1,544,241 ± 687,438 and 1,106,822 ± 560,821 (mean ± SD), respectively. We have added this information to METHODS, page 18, line 16.*

Comment 2-B: For the mouse fecal samples subjected to 16S target sequencing, what was the sequencing depth for each sample? Did the authors use any rarefication procedure before beta diversity calculation and taxonomic profiling?

*Response: The average number of reads per sample after quality control was 96,311 ± 35,047 (mean ± SD). We have added this information to METHODS, page 19, line 22. Before β-diversity analysis we checked minimum read depth of our samples by using the “biom summarize-table” command and set appropriate sequence depth to do βdiversity analysis. We did not do rarefaction analysis before taxonomic profiling, because the difference in sequencing depth was not large. According to previous literature, a 10-fold difference of sequencing depth requires rarefaction before taxonomic profiling (Weiss et al. Microbiome, 5:27, 2017), but this was not the case in our study.*

Comment 3-A: Engraftment of donor microbiota in this model does not appear to be robust (Additional Fig. 1).

A. Although in the supplemental material figure 1 post FMT did not group closer to the pre FMT fecal samples, they did not group closer to the donor fecal samples either, suggesting that both human and mouse are impacting the post FMT gut microbial community. Furthermore, regardless of the donor health status, the majority of the mouse post FMT samples clustered closer. Therefore, the "community microbiota uniquely dependent on the human subjects' flora" statement is not appropriate. Statistical analysis was not provided to support this statement.

*Response: We agree that murine microbiota derived from HD feces and IBD feces generated mixed clusters in PCoA analysis in new Additional Figure 1A. However, mouse microbiota derived from a single donor closely clustered even 28 days posttransplantation and segregated from the microbiota derived from other donors with significant statistical difference by PERMANOVA, as shown in new Additional Figure 1C.Although mouse microbiota were not complete restoration of human microbiota, post-transplantation-microbita was the subject-unique flora. Therefore, by analyzing the mouse microbiota after fecal transplantation, we could find association between colitis and specific microbe.*

*We added new PCoA results in Additional Figure 1 and mentioned them in RESULTS, page 6, line 17.*

Comment 3-B:B. Another concern here to be discussed is the potential to miss key strains that do not engraft. Furthermore, although Enteroccocus in enriched in UC transplant compared to HD (Fig. 4A), it is not clear if this is donor-derived E. faecium or the strain assignment (as per Fig. 6E) of the engrafted E. faecium strains.

*Response: We confirmed that no Enterococcus was present in the feces of mice before transplantation of human feces or inoculation of a single E. faecium strain, as shown in Additional Figure 4 (fecal transplantation) and Additional Figure 6 (single strain inoculation). Therefore, the Enterococcus that appeared after transplantation or inoculation were derived from either feces or the inoculated E. faecium strain.*

*We agree that we may miss key strains that were not engrafted by fecal transplantation. However, in metagenomic analysis, E. faecium was the most discriminative species between UC and HD. Based on our metagenomic results, we decided to examine the role of E. faecium, rather than other species.*

*We describe the methods and results of culture experiments in METHODS, page 21 line 20 and RESULTS, page 8, line 7 and lines 29, and page 9, line 18.*

Comment 4-A: It is interesting that Enteroccocus abundance correlates with cytokine production in the IL10-deficient mice (Fig. 4B). A. The median percentage abundance of 0.003% seems very low. Is there a linear correlation of Enteroccocus with cytokine production to help substantiate the potential correlation?

*Response: There was no linear correlation between the percentage Enterococcus present and cytokine production, but non-linear correlation demonstrated by Spearman’s rank correlation is shown in new Figure 4B. The individual coefficient r and P-value are shown in Additional Table 7. Please also see METHODS, page 22, line 3, and RESULTS, page 8, line 13. We deleted the old Figure 4B and created new Figure 4B showing correlation data.*

Comment 4-B:B. Is it clear that Enterococcus abundance maps to E. faecium? Does this association hold at the taxonomic level assignment (as done in Fig. 1A)?

*Response: We identified copy numbers of E. faecium and E. faecalis, representative Enterococcus strains in clinical patients, in mouse feces after transplantation of human feces by using quantitative PCR methods. E. faecium was detected at a higher level in UC compared with HD, but E. faecalis was not (see Additional Figure 5). However, copy number of E. faecium or E. faecalis alone did not correlate with colonic cytokine expression in mice, except for Il17a (Figure 4B). It was previously reported that E. faecalis promotes colitis in Il10−/− mice. We found that summing the copy number of E. faecium and E. faecalis showed a significant positive correlation with pathology score, and Il1b, Il6, Il17a and I12b expression, as shown in Figure 4B. Therefore, the overall abundance of the genus Enterococcus genus reflected abundance of E. faecium and E. faecalis, both of which seemed to contribute to colonic cytokine production. Please see METHODS, page 20, line 20, and RESULTS, page 8, line 15.*

Comment 4-C: C. What samples does the N=20 in Fig. 4B refer to? Is this individual mice or individual donors? Is this analysis performed with UC and/or CD donors? What is the effect of enterococcus abundance on cytokine production in HD colonized mice?

*Response: We chose a representative mouse from each mouse group to analyze the association of microbiota and cytokine expression. N = 40 (20 for low, 20 for high Enterococcus in the previous Figure 4B) referred to 37 mice group that received feces from 37 subjects (13 HD, 16 UC and 8 CD) and 3 VCM/DRPM controls in 3 independent experiments. We reduced the mouse group from 40 to 38 to analyze the correlation shown in the new Figure 4B because the VCM/DRPM groups overlapped. Thus, in the correlation experiment in new Figure 4B, microbiota, pathology, and cytokine data for 37 representative mice and 1 VCM/DRPM mouse were analyzed. It was difficult to analyze the effect of Enterococcus abundance on cytokine production in HD-colonized mice because the HD group contained only 13 members.*

Comment 5-A: The authors show a differential effect of IBD patient-derived and ATCC E. faecium compared to HD-derived E. faecium in Figure 5.

A. Can the authors confirm colonization (equivalent colonization) of the HD and IBD isolates to assess that this is not reflective of colonization? This should be provided for all isolate colonization experiments.

*Response: We confirmed colonization of the E. faecium strain, as shown in Additional Figure 6.*

Comment 5-B: B. Assuming equivalent colonization, can FISH be used to assess spatial location as potential mechanism for these strain differences.

*Response: Colonization was almost equivalent, as shown in Additional Figure 6. We hypothesized that the potential mechanism of the strain differences in Figures 5 and 6 might be the ability of the “inflammatory” strain to modify bile acid production, as shown in new Figure 7B. Therefore, we did not perform FISH, but instead discuss bile acidrelated promotion of colitis in the DISCUSSION, page 15, line 6.*

Comment 5-C: C. The data indeed suggest that E. faecium plays a role in disease, however the microbiota depleted IL10-/- model does not provide enough evidence to use the "causal" statement. Mouse microbiota can still contribute to the disease phenotype observed in the IBD recipient mice, especially due to the data shown in supplemental figure 1, in which pre FMT microbiota of mice receiving feces from healthy donors seems to be distinct from that of IBD recipient's mice. These points should be included in the Discussion.

*Response: We performed transplantation of HD55 feces or IBD51 feces, or inoculation of E. faecium strain IB51a into germ-free Il10−/− mice to create gnotobiotic mice. As a result, IBD51-transplanted and IB51a-colonized mice suffered more severe colitis than HD55-feces-transplanted mice (new Figure 6E). In terms of cytokines, IB51a-colonized mice showed increased expression of Tnf, Il1b, Il17a and I12b, whereas IBD51- transplanted mice only showed increase of Il23a compared with HD55-fecestransplanted mice (new Figure 6F). We are at least able to state that UC-derived E. faecium strain IB51a promotes colitis in a genetically susceptible host. We agree that mouse microbiota can still contribute to the disease phenotype in fecal-recipient-mice who whose microbiota was depleted by antibiotics.*

*We describe the gnotobiotic experiments in RESULTS, page 10, line 5, and also discuss the limitations of microbial depletion by antibiotics followed by fecal transplantation in the DISUCUSSION, page 14, line 14.*

Comment 6: The dichotomy between weight loss / pathology and cytokine production is a bit unclear in both Figures 5 and 6. For example, why does IBD36 derived strain and ATCC strain induce pathology and weight loss but differences in cytokine? Is this related to IL10-deficiency mechanistically? An additional model of colitis to evaluate these strains would help the authors assess this.

*Response: As pointed out, mice with pathological colitis did not necessarily show the same cytokine production pattern. Because single bacterial species or strains cannot explain disease occurrence or severity of IBD, multiple bacterial species or strains seem to contribute to the pathology of IBD colitis. Differences in cytokine production may be caused by difference in bacterial composition. Actually, gnotobiotic IB51a-colonized mice showed different a cytokine pattern from IBD51-feces-transplanted mice (Figure 6F). We discuss this point in the DISCUSSION, page 13, line 23. Creating an additional colitis model would be ideal, but to start it we would need at least 3 months for approval of animal experiments and preliminary experiments, and more time to obtain final results. We think creating such an additional model should be future work, so we refer to it in the DISCUSSION, page 14, line 13.*

Comment 7-A: The hypothesis that strain variation account for differences in intriguing, but not strongly supported by the data (as stated on page 10, line 5-6).

A. The resolution of figure 6 is very low making it difficult to read strain names. It is also difficult to decipher strain nomenclature.

*Response: The low resolution might be caused by compression of the figure in the upload process of the manuscript. The original figure was made with sufficient resolution (600 dpi). However, we have now increased the resolution of Figure 6 to 800 dpi.*

Comment 7-B: B. While Fig. 6E supports the possible existence of genomic strains, the correlation with colitis pathology and/or cytokine production is confusing. While pathogenic ATCC isolates segregates with 51a, disease inducing isolate 18a segregates with probiotic and HD strains. Moreover, HD59a (unclear due to low resolution of the figure provided) segregates with the ATCC isolate. These data do not clearly support a meaningful association with function. Can the authors clarify this? Mechanistic data showing a strain effect (suggested in the Discussion) would be ideal. It would be helpful at least to provide colitis and cytokine data for each isolate profiled in Fig. 6E as well as confirmation of colonization.

*Response: We additionally examined pathology of and cytokine production in IB44ainoculated and SF68-inoculated mice, and show the data in Additional Figure 7. Colonization was confirmed as shown in Additional Figure 6. SF68, an E. faecium strain commercially used as a probiotic, resulted in little inflammation and cytokine production in the colon, but UC-derived strain IB44a resulted in a significantly increased pathology score and expression of Tnf, Il17a and Il12b compared with negative controls. These findings are in RESULTS, page 10, line 1.*

*We agree that, like HD26a and SF68, strain IB18a was clustered in the “probiotic” cluster. Because strain IB18a resulted in less cytokine production than inflammatory strain IB51a, IB18a may be an intermediate phenotype between probiotic HD26a and inflammatory IB51a. We identified that the “inflammatory” cluster was enriched in (KEGG pathway) secondary bile acid biosynthesis, as shown in Figure 7B. We would like to elucidate why IB18a in the “probiotic” cluster shows a partially inflammatory phenotype in a future project. Such work will require trial and error in gene-deletion experiments in the particular strain and subsequent colonization experiments in animals, so it will take years. In this study, we just discuss possible mechanisms related to secondary bile acid biosynthesis in the DISCUSSION, page 15, line 6.*

Comment 7-C: C. Error bars in Figure 6C seem larger than would be expected with 2 and 3 star p-value significance. Can the authors clarify this finding? Did the authors apply any multiple comparison correction in the analyses described in figure 6?

*Response: We apologize. The error bars in Figure 6C were SD, not SE, so looked larger than expected. We have amended the error bars. We also performed multiple comparison correction of the data in Figure 6 by using the Kruskal Wallis test followed by Dunn’s test. We amended the significance in Figure 6 and changed the RESULTS, page 9, line 19 and line 27, according to the new statistical analysis.*

Comment 8-A: The authors suggest that E. faecium presence correlates with disease extent and combination therapy.

A. For the N=5 with E. faecium, does E. faecium abundance linearly correlate with disease activity score?

*Response: At first we examined copy number of E. faecium in human feces (per gram) by quantitative PCR, a more sensitive method than metagenomic sequencing (please see Figure 1E). Then, the number of UC patients in which E. faecium was detected thus increased from 5 to 9. As shown in Additional Figure 10, the presence of E. faecium was not associated with Mayo score.*

*We have modified RESULTS, page 11, line 12.*

Comment 8-B: B. For the N=5 with E. faecium, does genomic analysis suggest a particular strain type?

*Response: By PCR, E. faecium was detected in 9 UC patients, but not all E. faecium strains were isolated from those 9 patients.*

Comment 8-C: C. It is interesting that the authors focused on E. faecium finding if only present in 5/16 UC patients. What is driving colitis in 11/16 samples if not E. faecium (in Fig. 2A)? Also, what is the source of the Enterococcus seen in Figure 4, particularly for the samples from donors without Enterococcus?

*Response: In humans, not only microbial composition, but also treatments, determine colitis activity. Thus, to answer the comment we focus on the transplanted mouse experiments in which we simply assessed the effect of microbial composition on colitis. As described in our response to Comment 4B, in E. faecium-absent mice, E. faecalis seemed to have an inflammatory effect on the colon. The presence of E. faecalis significantly correlated with expression of Il17a in the mouse colon. The summed copy number of E. faecium and E. faecalis positively correlated with pathological inflammation and production of several cytokines. In humans, it is presumed that other bacterial species may drive colitis in E. faecium-absent patients. By doing sensitive detection by qPCR, we found that after transplantation of feces from donors without E. faecium, no E. faecium was found in the mouse microbiota. Therefore, we can state that the origin of mouse E. faecium was donor feces.*

**Second round of review**

**Reviewer 1**

All questions answered.

**Reviewer 2**

This is a revised manuscript from Dr. Mizukoshi and colleagues.  The revisions are responsive to the critiques and I believe the manuscript is strengthened significantly.  The authors have added details in the supplemental data, which I think are very helpful in providing a complete evaluation of the work.  

There are several minor additional points that I believe could be addressed:

1.  The relative abundance of E. faecium in the initial submission appeared very low but it is hard to tell from these data.  I think clarification of the potential effect of these low abundant Keystone species (possibly via indirect metabolic effect) could be discussed.

2.  A major finding in this paper is the potential for strain variability and particularly bile acid metabolic pathways in colitis pathology (Fig. 7B).  Although the authors state that it would take too long to evaluate genetic mutants, in vitro analysis of these metabolic pathways (or ex vivo in colonized animals) would be helpful in confirming the functionality of this strain variability.

**Authors Response**

**Point-by-point responses to the reviewers’ comments:**

*Response to Reviewer 2: Thank you for your review of our paper. We have answered each of your points below. Line numbers refer to the new version of the paper. First of all, we need to apologize our mistake in Figure 7B.*

*In old Figure 7B, ko pathway number was correct, but pathway name was switched in each other. We amended the mistake in new Figure 7B. Because ko pathway number was correct, conclusion is the same as previous version. We checked all data again, and there is no mistake in the finally revised version of manuscript.*

Comment 1: The relative abundance of E. faecium in the initial submission appeared very low but it is hard to tell from these data. I think clarification of the potential effect of these low abundant Keystone species (possibly via indirect metabolic effect) could be discussed.

*Response: E. faecium strains in the inflammatory cluster possessed bile salt hydrolase (BSH) activity while 2 strains in the probiotic cluster, HD26a and HD50a, did not possess the BSH activity as shown in new Additional Figure 10A. In addition, reactive oxygen species (ROS) in culture medium generated from inflammatory E. faecium strains was higher than probiotic strains as shown in new Additional Figure 10B and C. Thus, metabolites produced from inflammatory E. faecium strains might affect colon pathogenesis and account for ability of low load of the strains to induce colitis in Il10-/- mice. According to Additional Figure 5, load of E. faecium in feces ranged from 102 to 108 copy number gram feces. Previous literatures (J Clin Microbiol 2011;49:3656-8 and Gastroenterology 2008;134:768-80) demonstrated that 104 to 108 CFU/gram feces of Clostridium difficile or 103 to 108 CFU/gram feces of Salmonella typhimurium caused infection and colitis. Bacterial load of E. faecium in mouse feces of this experiment was compatible to the load of other colitic bacteria. Taken together, E. faecium in feces of our mice experiment should be enough to induce colitis in the mice.*

Comment 2: A major finding in this paper is the potential for strain variability and particularly bile acid metabolic pathways in colitis pathology (Fig. 7B). Although the authors state that it would take too long to evaluate genetic mutants, in vitro analysis of these metabolic pathways (or ex vivo in colonized animals) would be helpful in confirming the functionality of this strain variability.

*Response: As answered in the comment 1, E. faecium strains had variability in activity of BSH and ability to generate ROS in culture experiment as shown in Additional Figure 10. Inflammatory strains possessed BSH activity, but 2 probiotic strains, HD26a and HD50a, did not. Role of BSH in pathogenesis in colitis has not been elucidated in this study as well as previous reports. However, different ability of bile acid metabolism in E. faecium strains may be involved in promotion of colitis because secondary bile acid biosynthesis following BSH reaction have important roles in colitis as discussed in DISCUSSION, page 16, line 8. In addition, increased ROS generation observed in the culture of inflammatory E. faecium strains might be related to colitis pathogenesis. Because lipoic acids are anti-oxidant, less abundance of pathway of lipoic acid metabolism in the inflammatory cluster may be associated with increased generation of ROS. Due to complexity of pathway of lipoic acid metabolism and limited time for the revision, we could not directly demonstrate association between increased ROS generation and metabolites related to lipoic acids. We would like to fully elucidate these unanswered points in the future project. Anyway, 10 E. faecium strains had variability in BSH activity and ability to generate ROS, so the variability may be associated with difference in colitis-promoting ability of the strains.*

*To answer the comment 1 and 2, we created new Additional Figure 10, and moved old Additional Figure 10 to 11. We added results and methods of culture-based experiments in RESULTS, page 11, line 8 and Additional METHODS, page 4, line 22. We also modified DISCUSSION, page16, line 14.*

*We wish to thank the Reviewers again for their valuable comments.*
